# Supplementary figures and images for: Loss of Mfn1 but not Mfn2 enhances adipogenesis
Source: PLoS One. 2024 Dec 31;19(12):e0306243. doi: 10.1371/journal.pone.0306243 (PMC11687706; doi:10.1371/journal.pone.0306243)

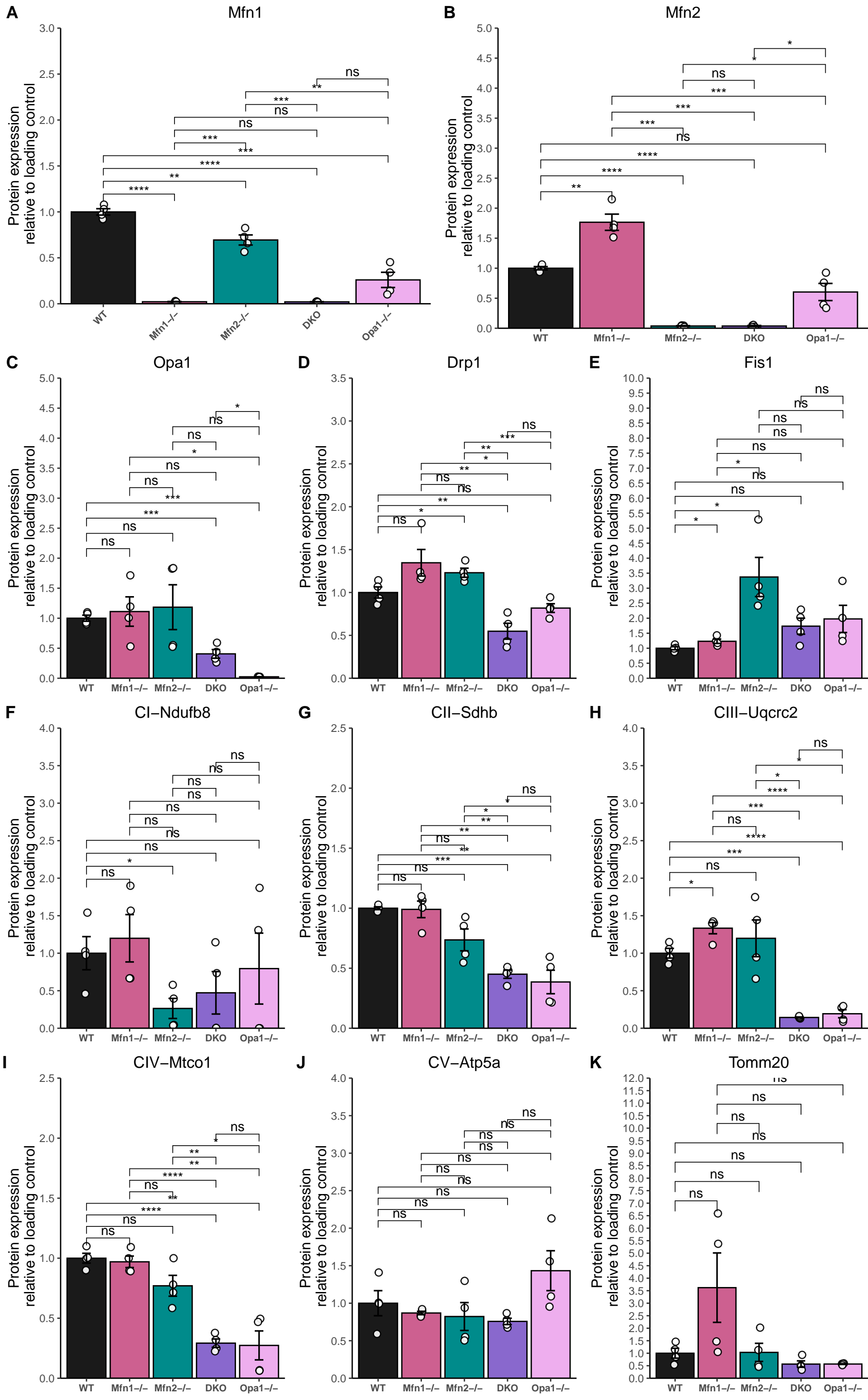

Supplement: S1 Fig — Quantification of protein expression (relative to wild-type) for all proteins shown in Fig 1A and 1F. Each data point represents a separate biological replicate. Stars indicate p-values following pairwise comparisons between groups: * p<0.5, ** p < .01, *** p < .001, **** p < .0001. ns, not significant. (PDF) [file pone.0306243.s001.pdf]

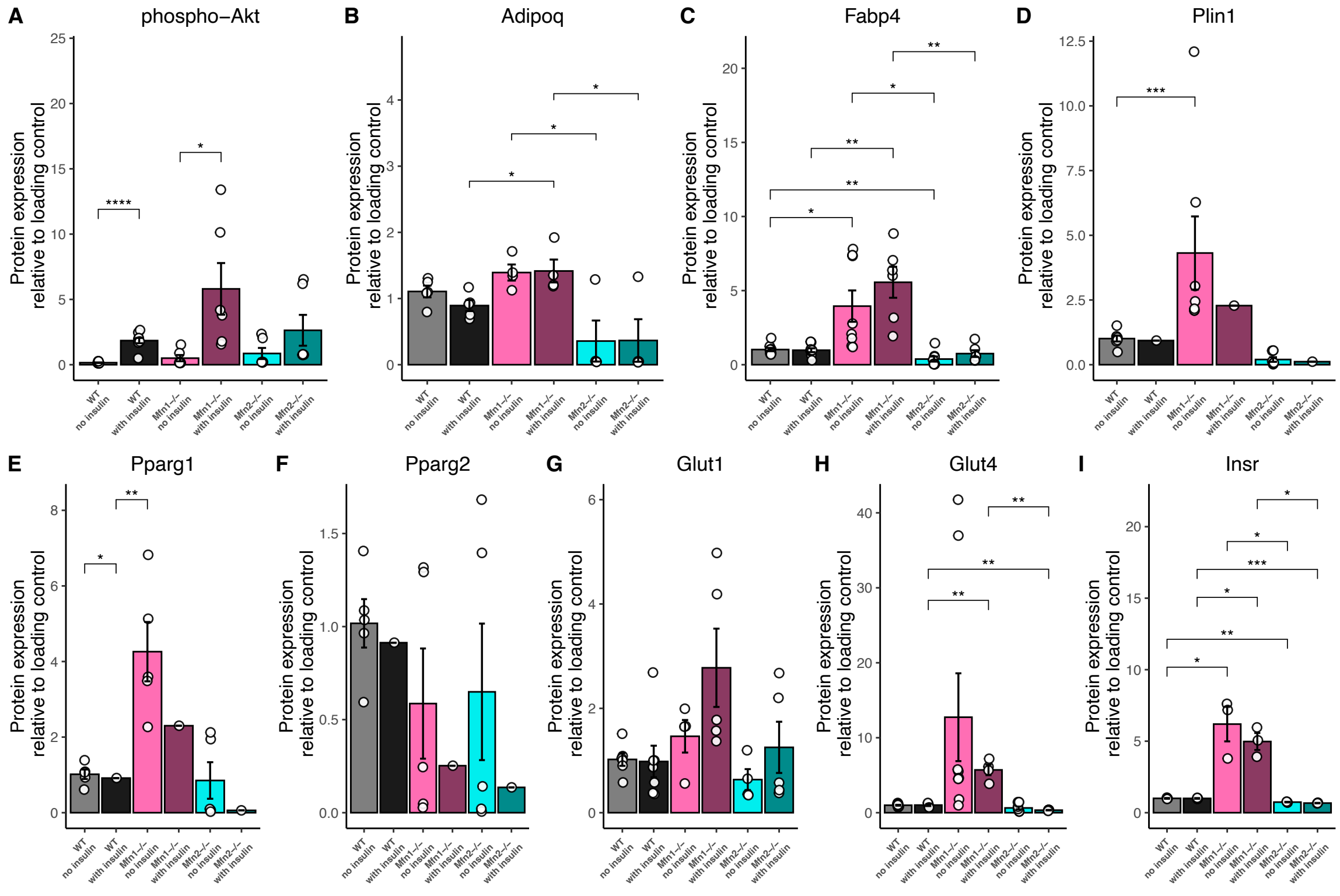

Supplement: S2 Fig — Quantification of protein expression (relative to wild-type) for all proteins shown in Fig 2D from knock-out MEFs. Each data point represents a separate biological replicate. Stars indicate p-values following pairwise comparisons between groups: * p<0.5, ** p < .01, *** p < .001, **** p < .0001. ns, not significant. (PDF) [file pone.0306243.s002.pdf]

## Day -2 pre-adipocytes

**A**

Wild-type    Wild-type +*Pparg2*    *Mfn1*<sup>-/-</sup> +*Pparg2*    *Mfn2*<sup>-/-</sup> +*Pparg2*

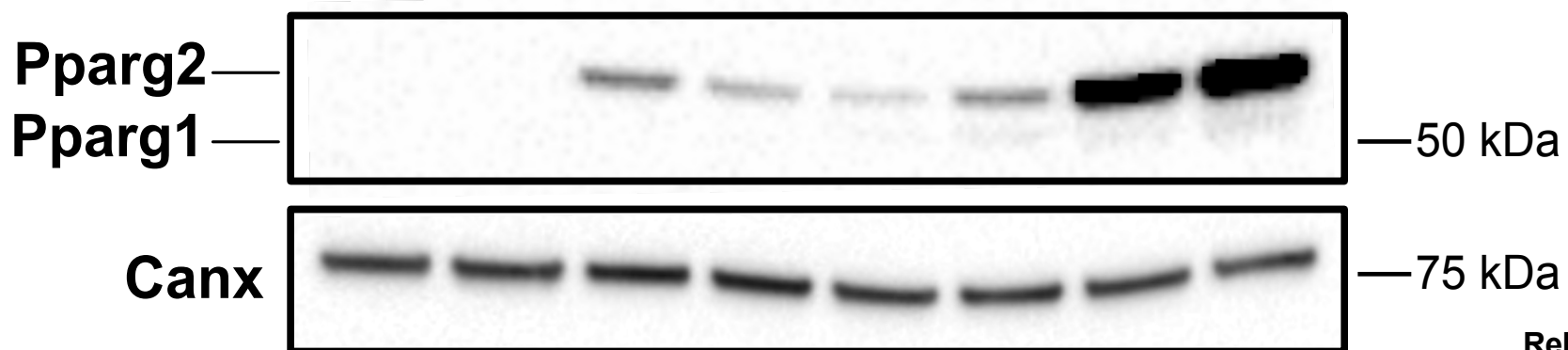

**B**

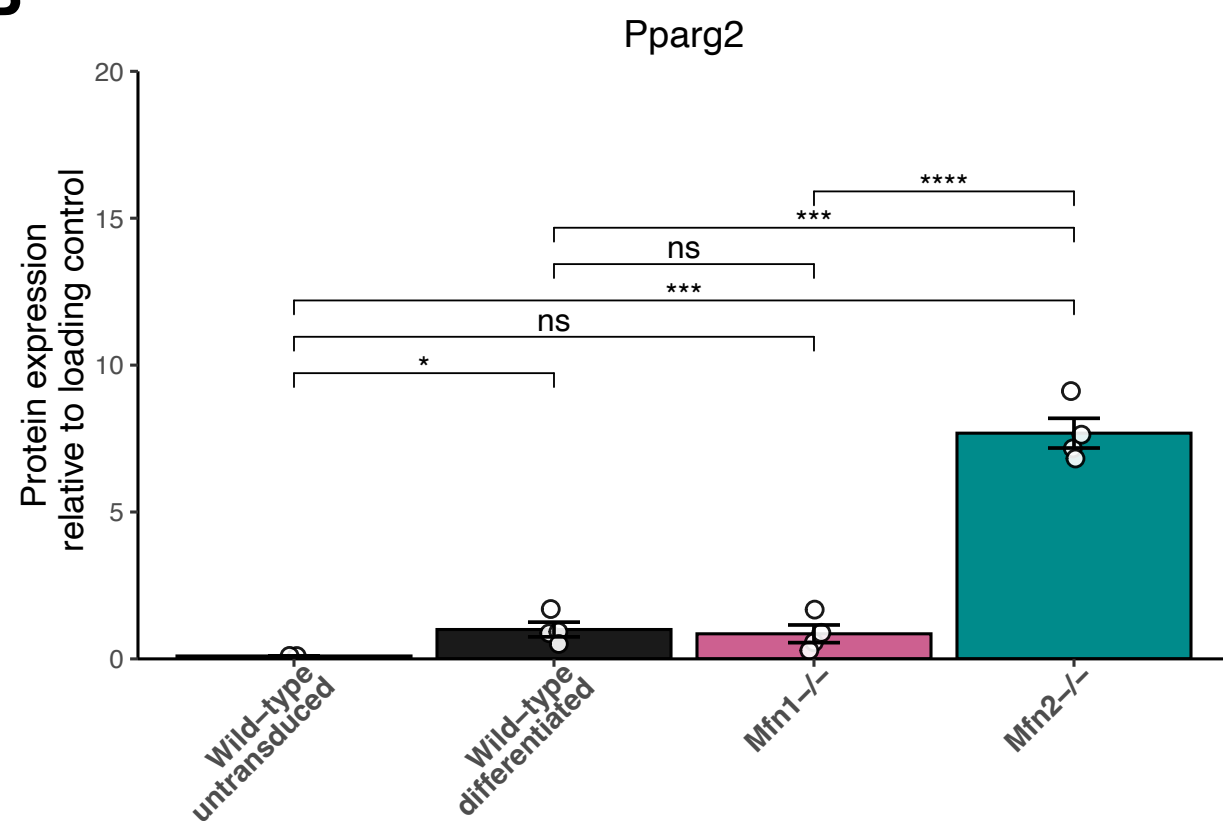

**C**

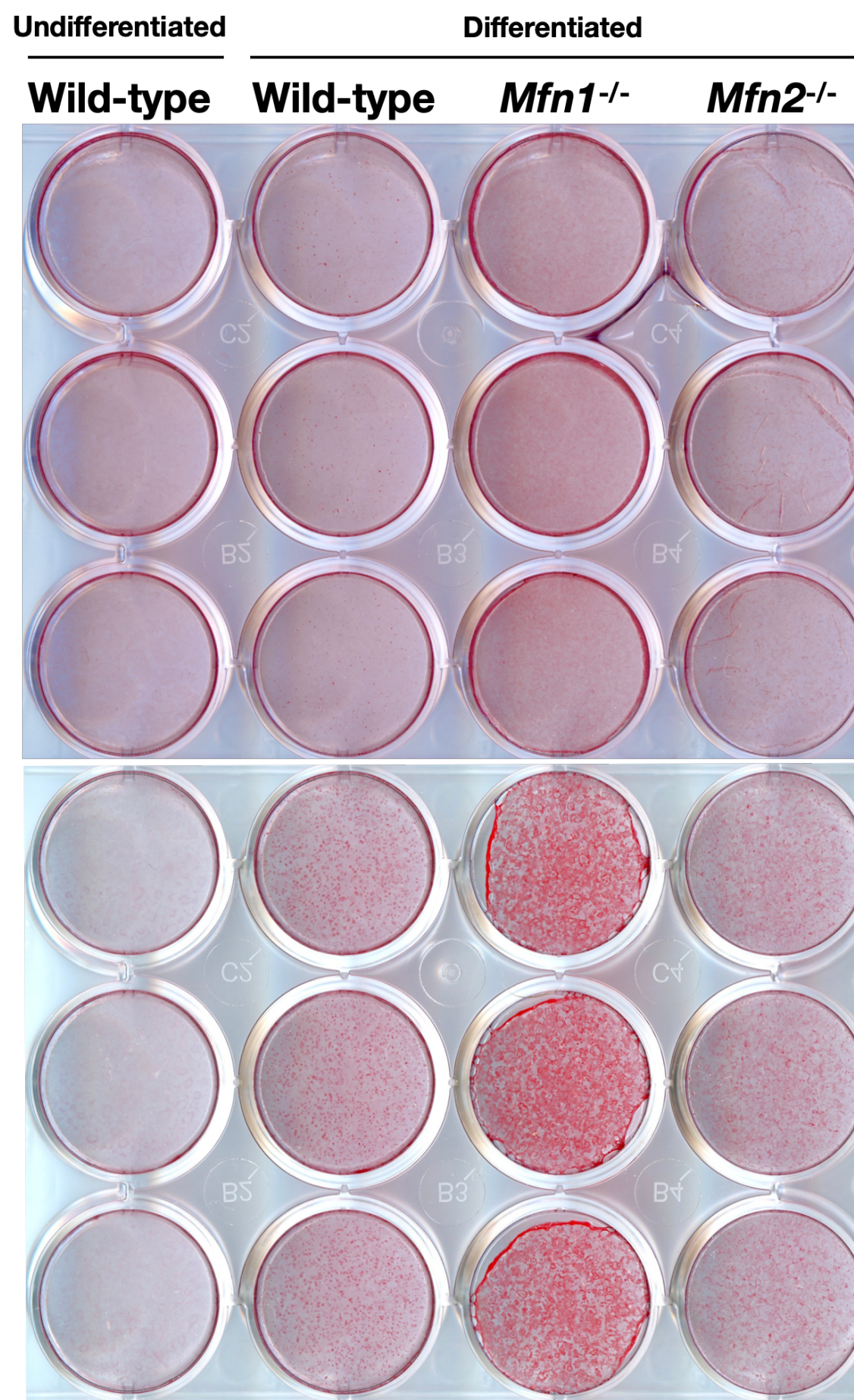

**D**

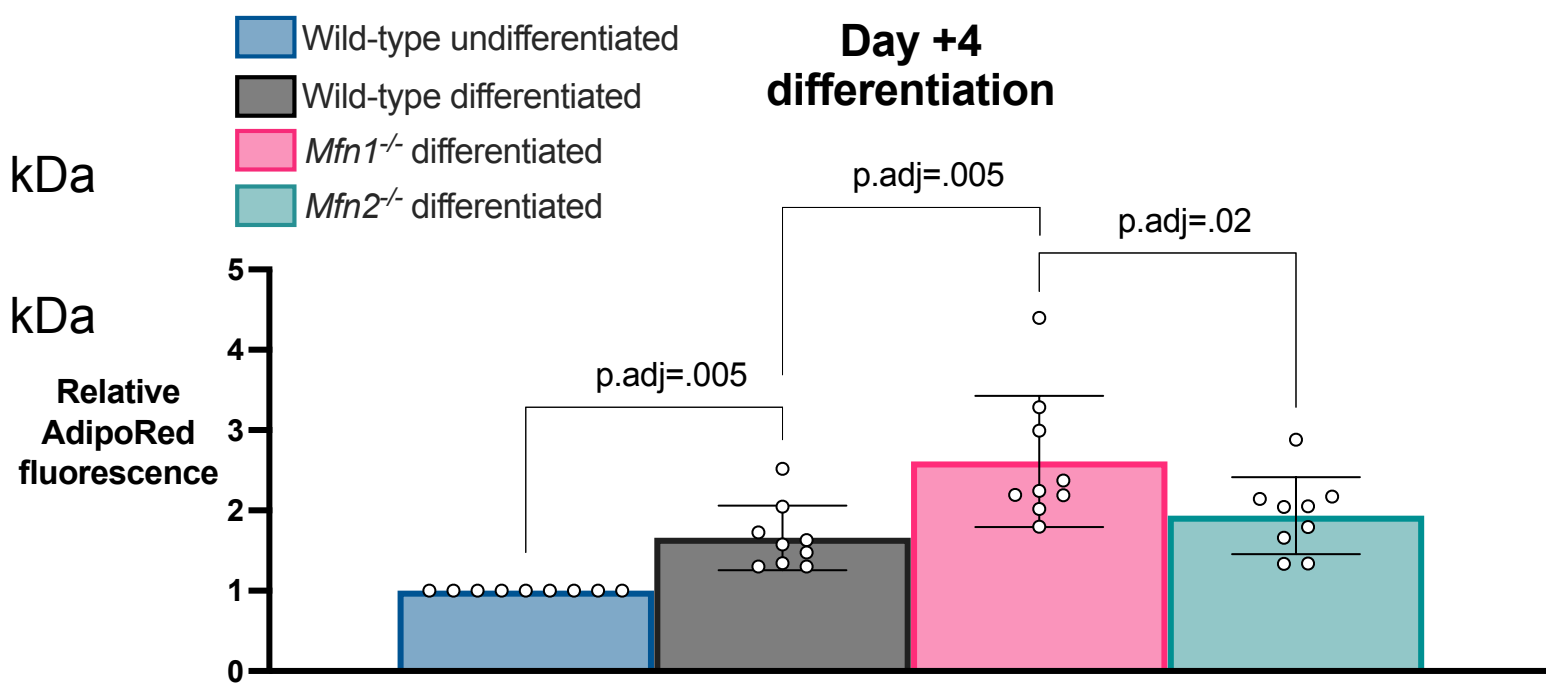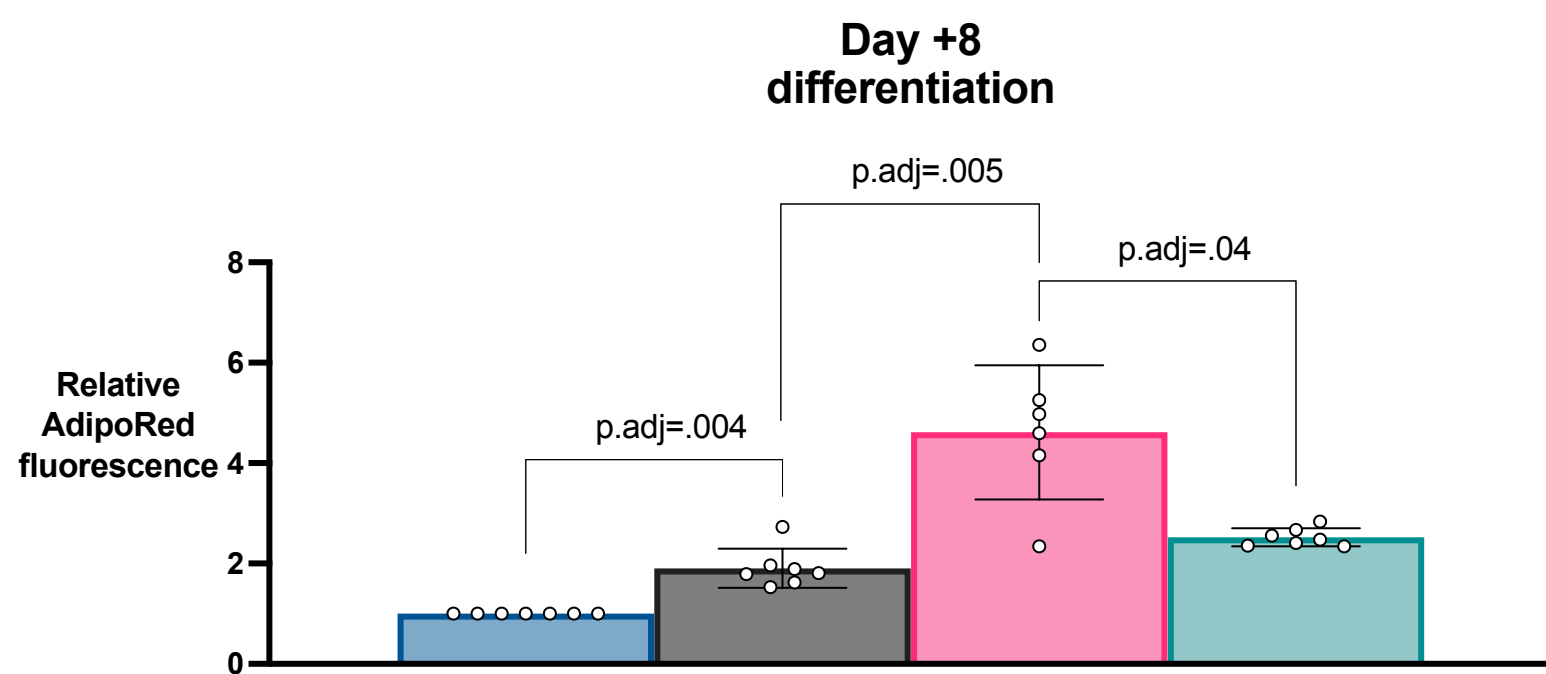

**E**

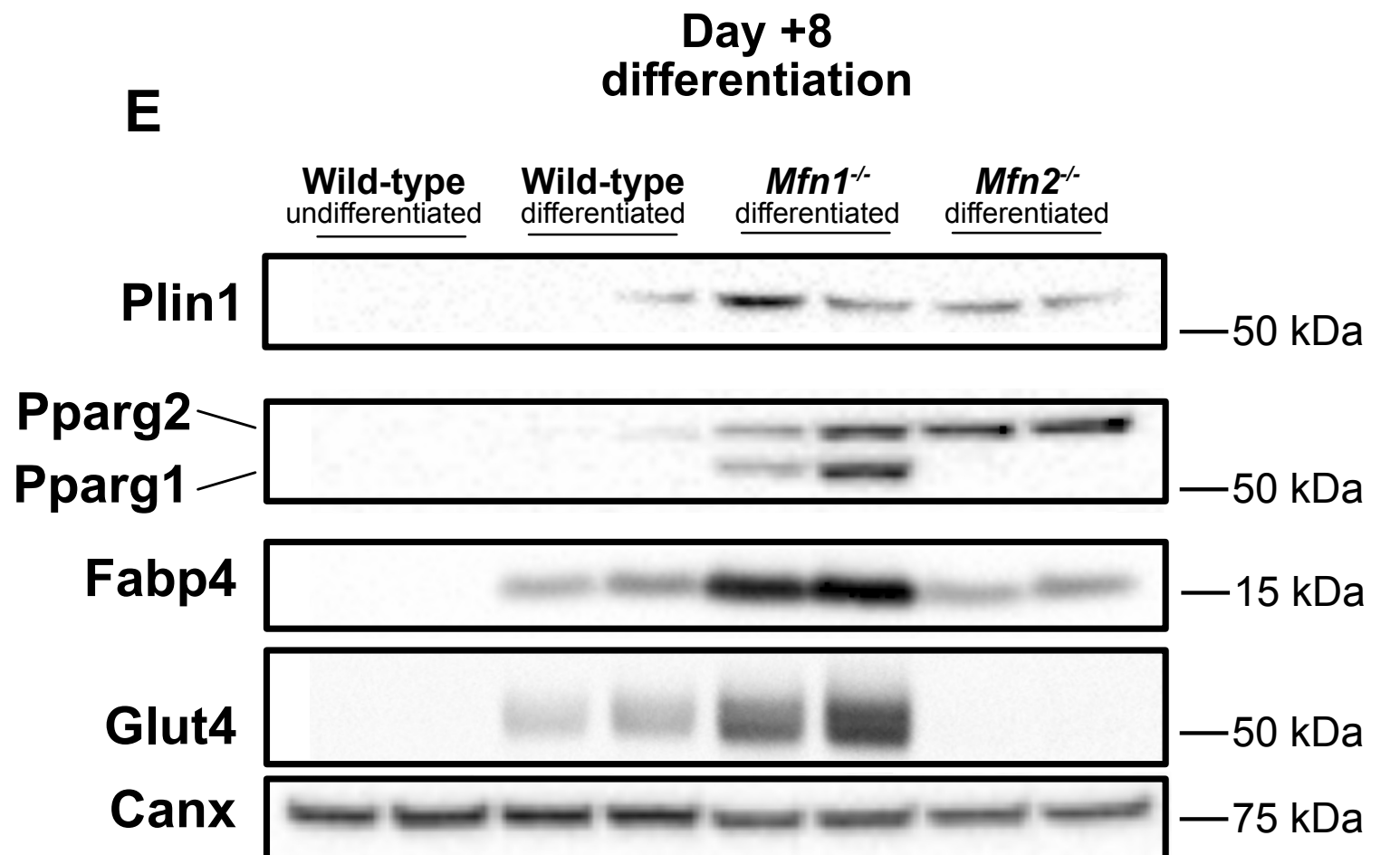

Supplement: S3 Fig — Pparg2 was over-expressed in MEFs to enhance their adipogenic differentiation capacity and cells were assessed on day +4 and +8 of protocols. (A) Western blot illustrating the relative Pparg2 over-expression in cell lines used compared to non-transduced wild-type cells. (B) Quantification of Pparg2 expression from the Western blot in panel (A). (C) Oil Red O staining differentiated MEFs compared to undifferentiated wild-type MEFs. (D) Fluorometric quantification of AdipoRed neutral lipid dye at day +4 & day +8. Each data point represents a separate biological experiment. Data is expressed relative to undifferentiated wild-type for each biological replicate. (E) Western blot for markers of adipocyte differentiation at day +8 from MEFs over-expressing Pparg2. All p-values represent pairwise comparisons between knock-outs and wild-type using t-tests, adjusted for multiple testing (p.adj). Data is representative of at least 3 independent replicates. (PDF) [file pone.0306243.s003.pdf]

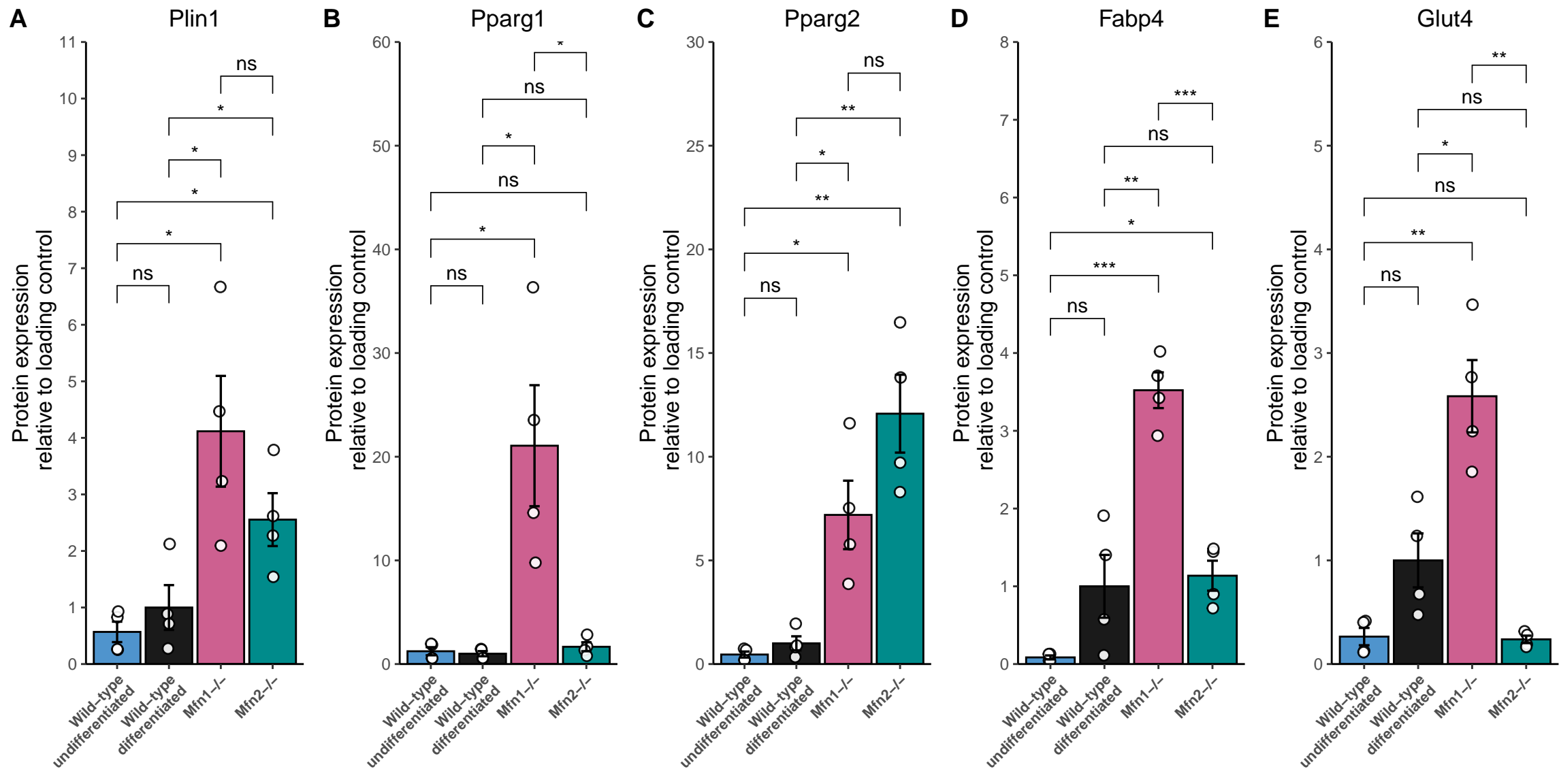

Supplement: S4 Fig — (A-E) Quantification of protein expression from knock-out MEFs over-expressing Pparg2, from S3E Fig. Each data point represents a separate biological replicate. Stars indicate p-values following pairwise comparisons between groups: * p<0.5, ** p < .01, *** p < .001, **** p < .0001. ns, not significant. (PDF) [file pone.0306243.s004.pdf]

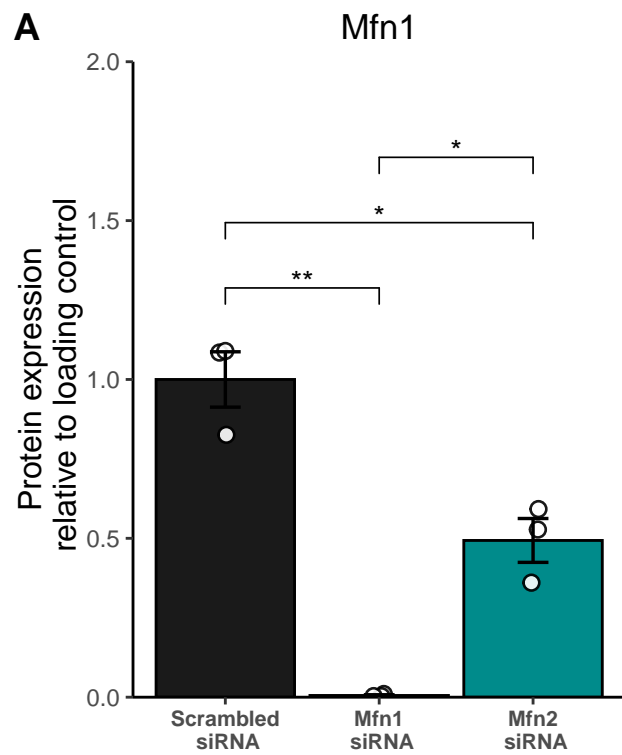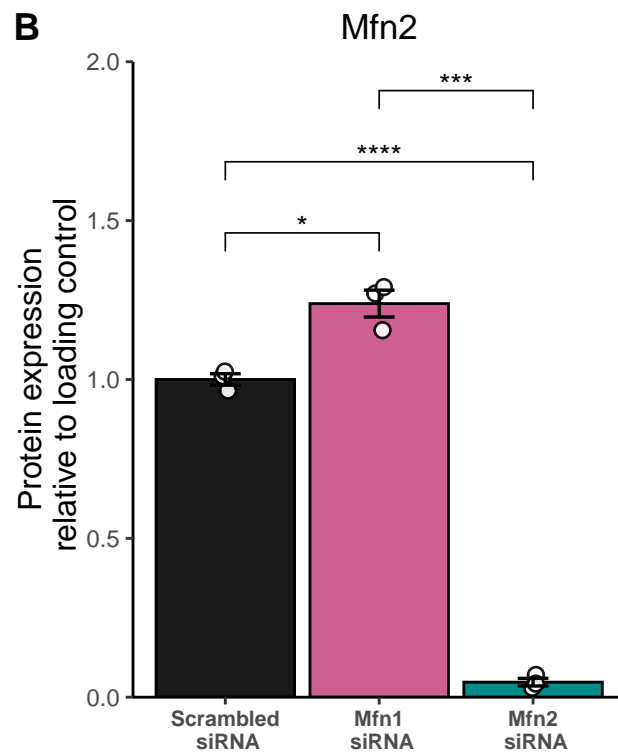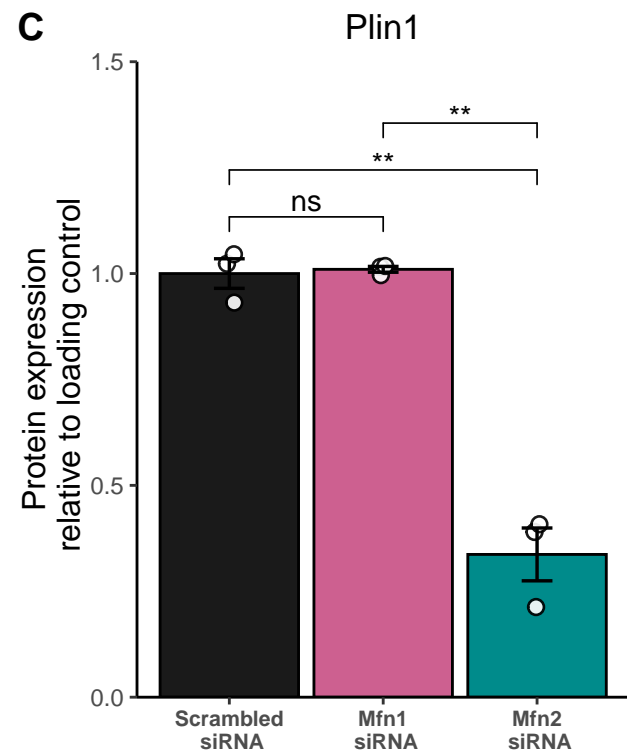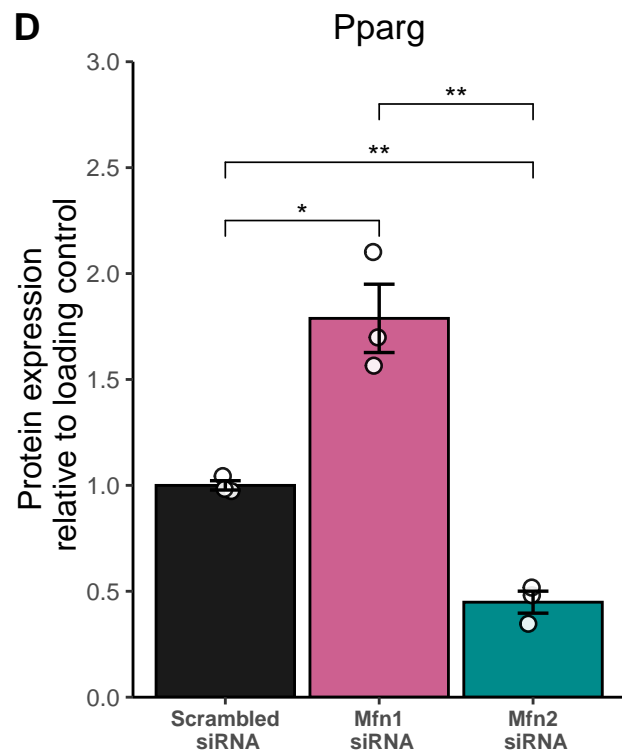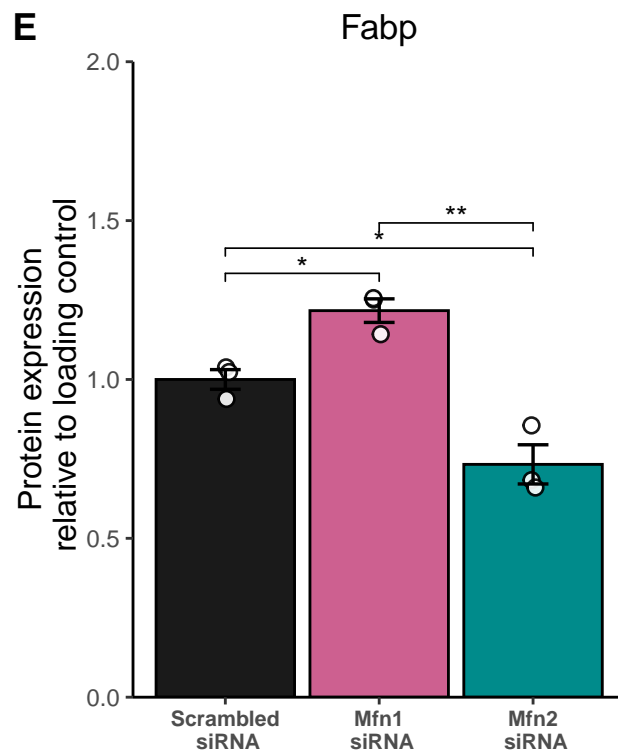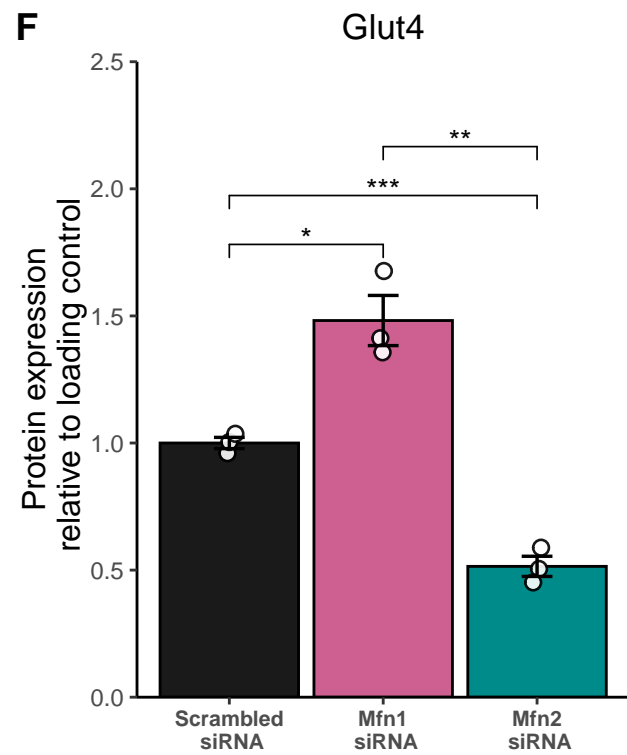

Supplement: S5 Fig — Quantification of protein expression (relative to scrambled control) for all proteins shown in Fig 4A. Each data point represents a separate biological replicate. Stars indicate p-values following pairwise comparisons between groups: * p<0.5, ** p < .01, *** p < .001, **** p < .0001. ns, not significant. (PDF) [file pone.0306243.s005.pdf]

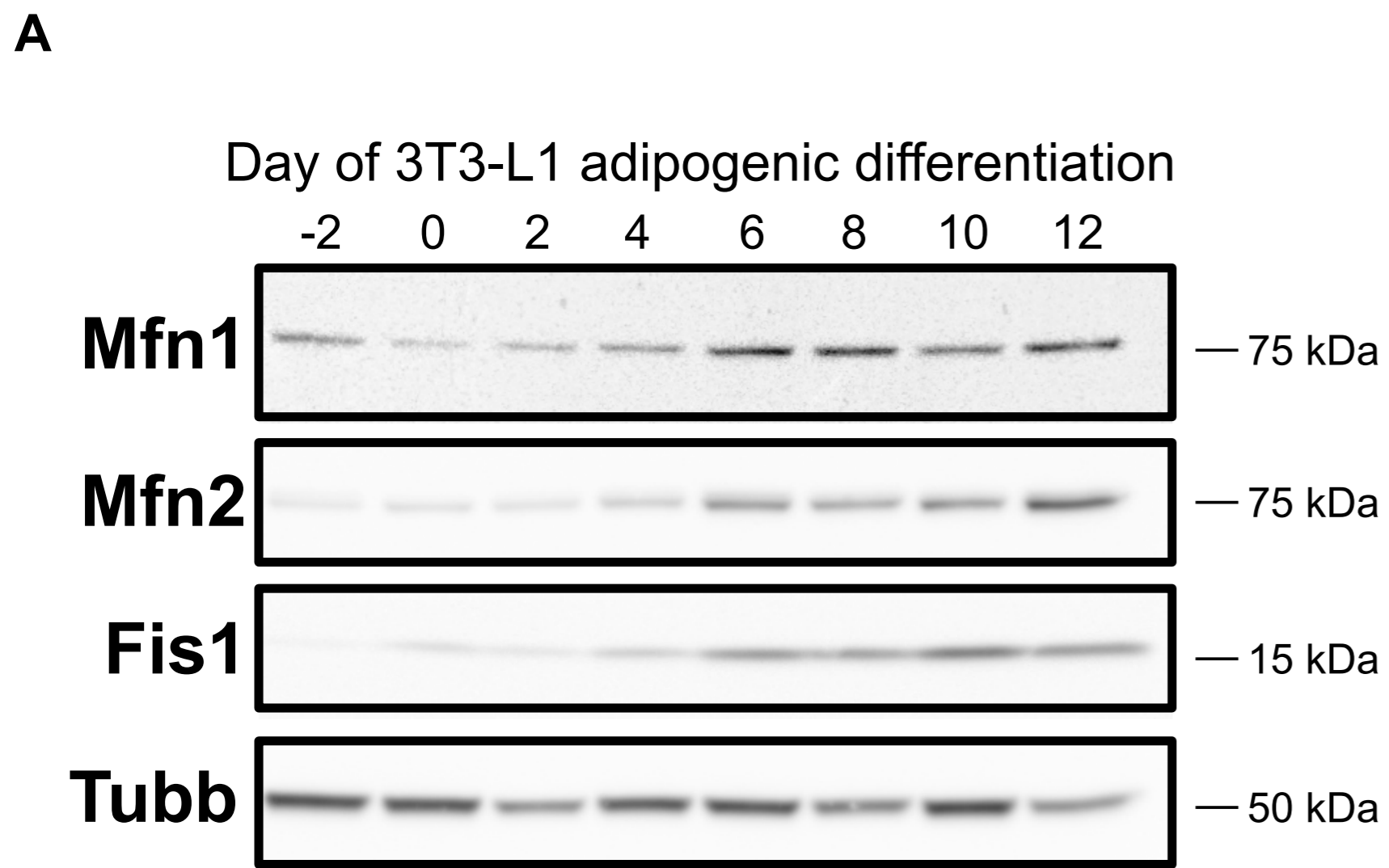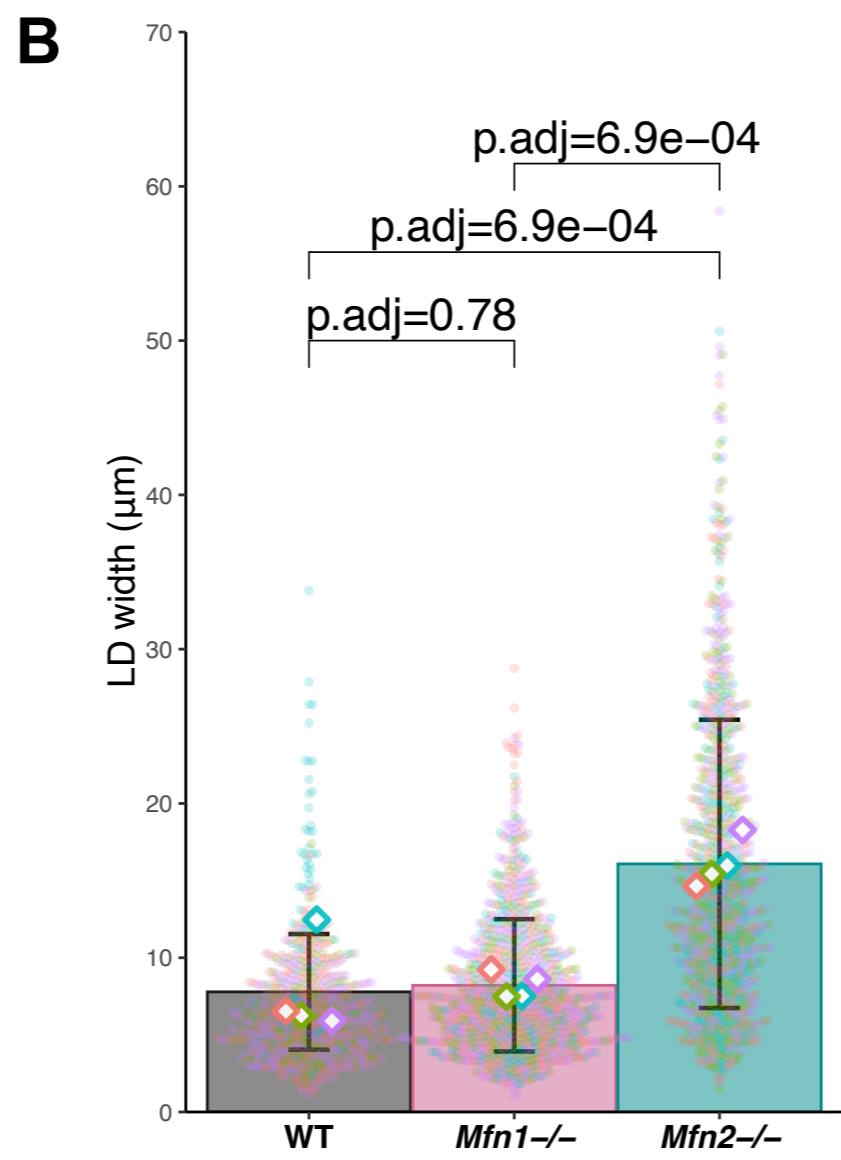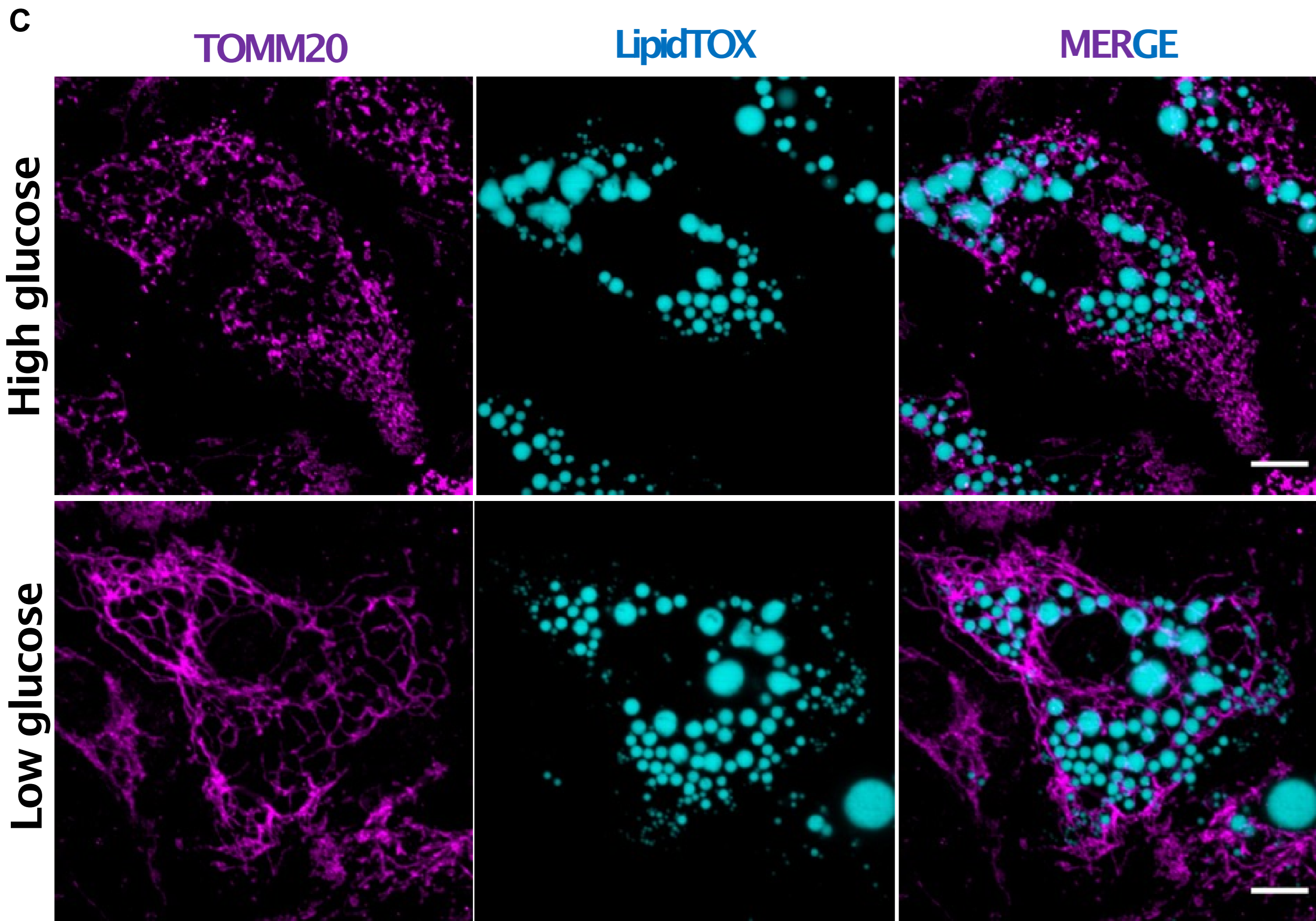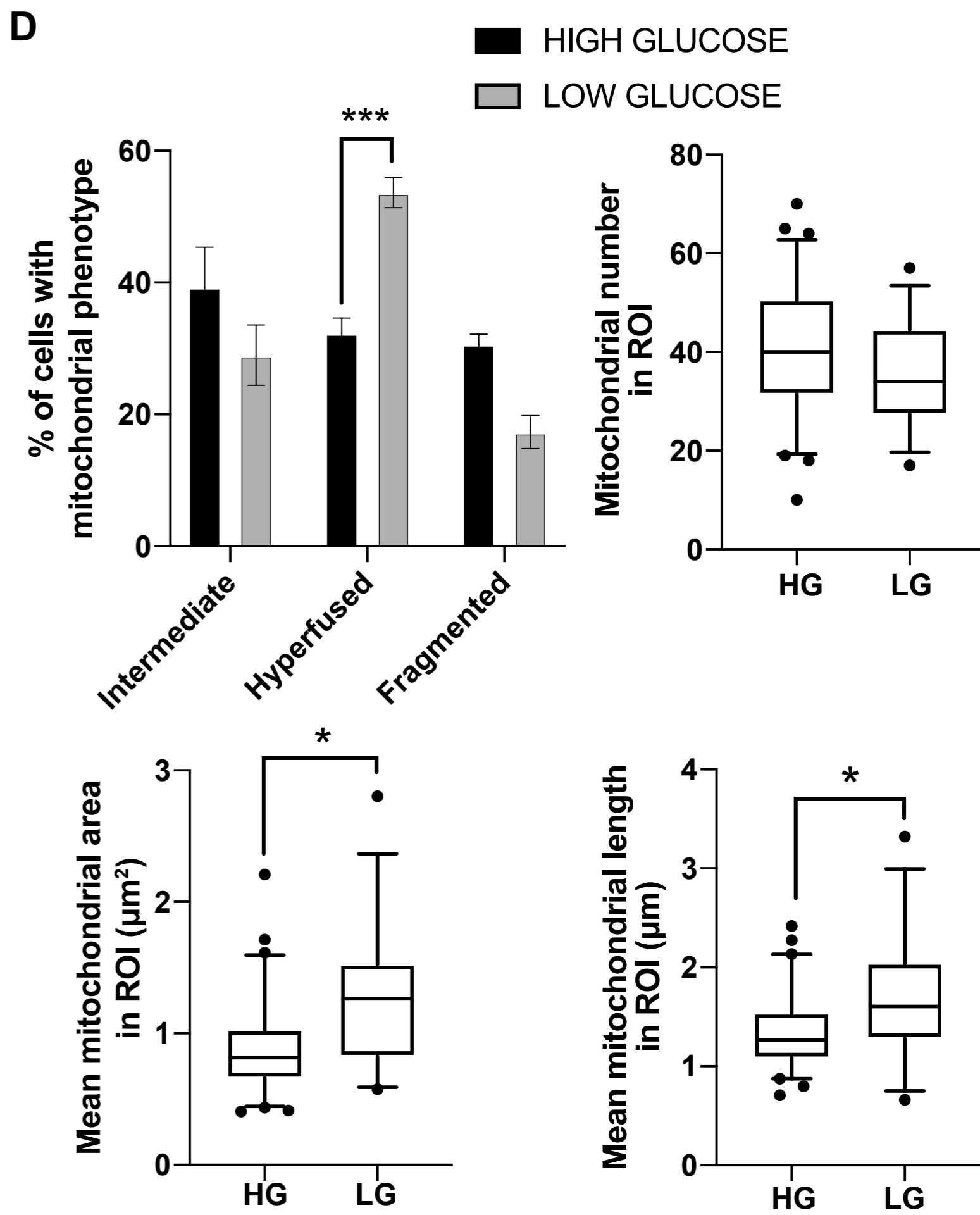

Supplement: S6 Fig — (A) Representative western blot showing change in mitofusin expression in 3T3-L1 cells during adipogenic differentiation. (B) Measurement of lipid droplet width from oil red O staining of day +12 3T3-L1 adipocytes treated with scrambled, Mfn1-targeted, or Mfn2-targeted siRNA on alternate days from day -2 to day +12 of differentiation. Data from four biological replicates. (C) Representative confocal images of 3T3-L1s cells at day +12 of differentiation under high glucose (HG) and low glucose (LG) conditions. Mitochondria were labelled using an anti-TOMM20 antibody (purple) and neutral lipids were stained with LipidTox (blue). Scale bars: 10 μm. (D) Quantification of mitochondrial morphology, and different mitochondrial parameters including number, area, and length in 225 μm2 region of interests from (A). Stars indicate p-values following pairwise comparisons between groups: * p<0.5, *** p < .001. (PDF) [file pone.0306243.s006.pdf]

A

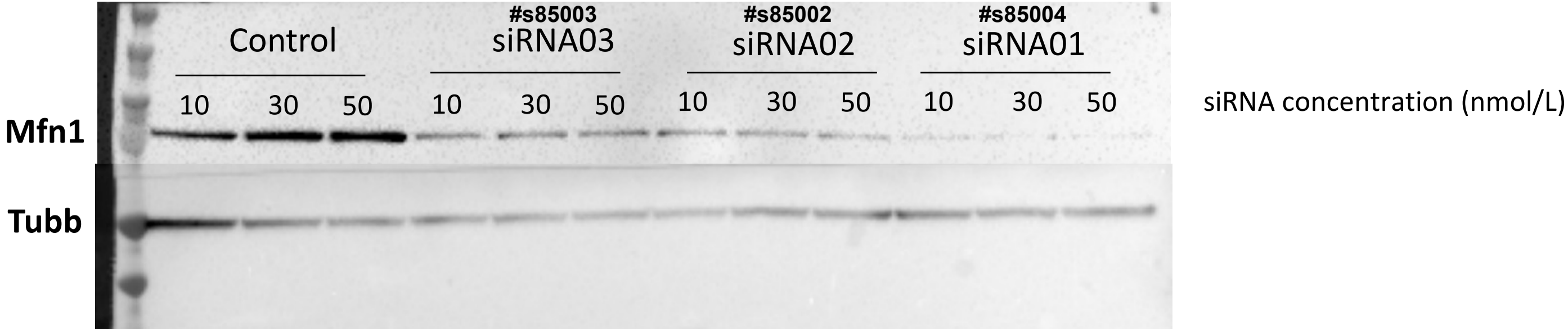

B

Mfn1 silencer select knock-down in differentiated (day +10) 3T3-L1

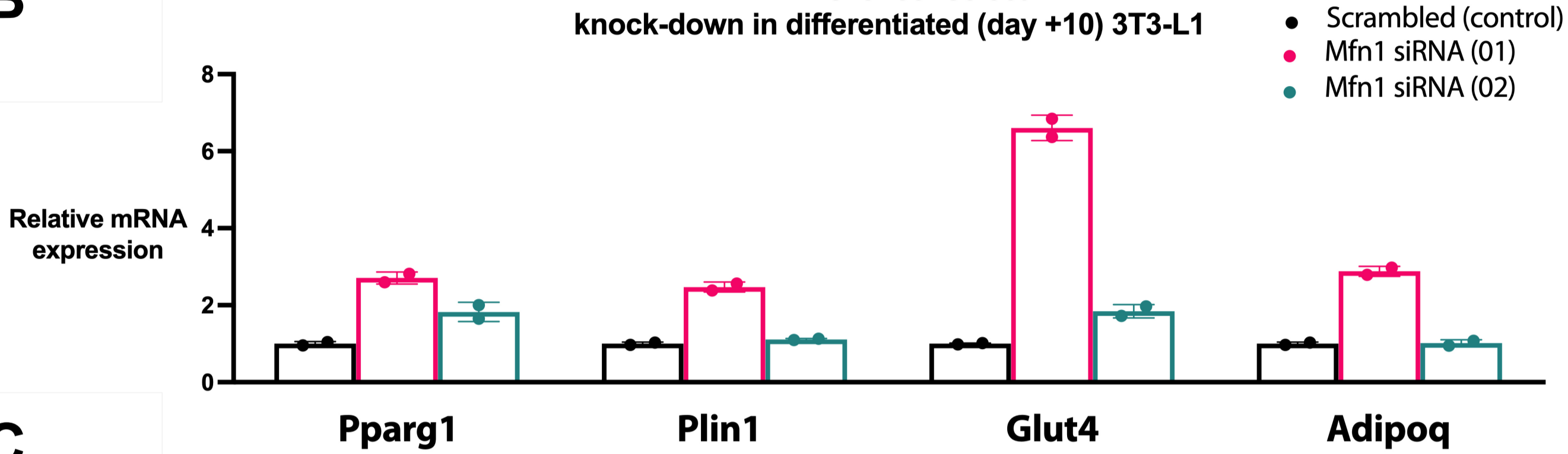

C

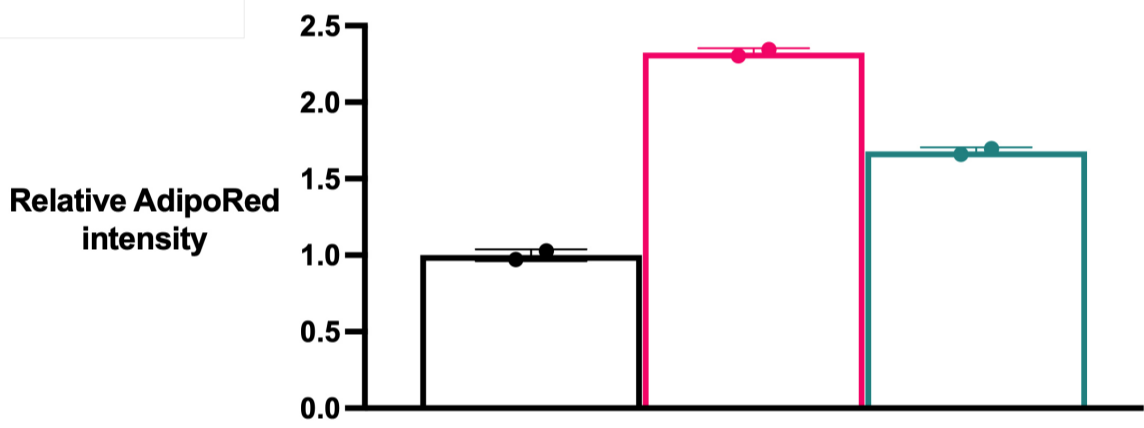

D

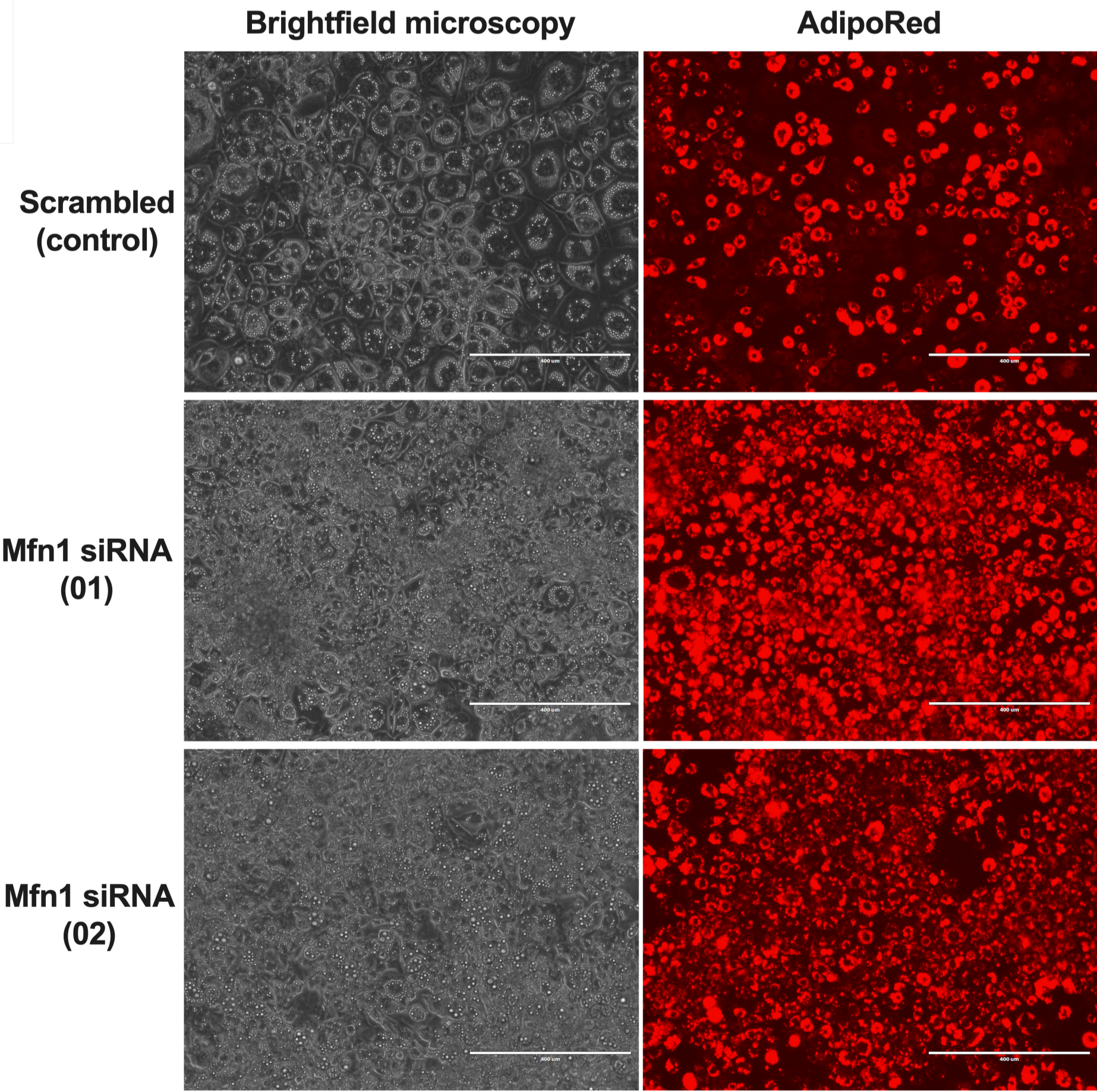

Supplement: S7 Fig — Undifferentiated 3T3-L1 pre-adipocytes were treated with three separate Invitrogen Silencer Select siRNAs (CatIDs: s85003 = ‘siRNA03’, s85002 = ‘siRNA02’, s85004 = ‘siRNA01’) for 24-hours, each siRNA was used at 10nmol/L, 30nmol/L, and 50nmol/L. A, Western blot demonstrating Mfn1 protein knock-down efficacy. Following this, 3T3-L1 adipocytes were treated with s85002 (= ‘siRNA02’) and s85004 (= ‘siRNA01’) until day +10 differentiation. B, Relative mRNA expression of markers of adipogenesis at day +10 differentiation. C, Lipid accumulation at day + 10 as measured by fluorescence using AdipoRed. D, Representative images for adipogenic differentiation using brightfield microscopy and AdipoRed staining. Data from n = 2 biological repeats. (PDF) [file pone.0306243.s007.pdf]
