# Supplementary material for: Loss of Mfn1 but not Mfn2 enhances adipogenesis
Source: PLoS One. 2024 Dec 31;19(12):e0306243. doi: 10.1371/journal.pone.0306243 (PMC11687706; doi:10.1371/journal.pone.0306243)
Supplement: S1 Table — List of all reagents and equipment used in this study. (DOCX) [file pone.0306243.s008.docx]

| **Reagent** | **Supplier** | **CatID** |
| --- | --- | --- |
| 2-mercaptoethanol | Gibco | 31350010 |
| 4-12% Bis-Tris gel | Invitrogen | NP0335BOX |
| AdipoRed | Lonza | PT-7009 |
| Alexa Fluor 488 | Invitrogen | A-11094 |
| BODIPY | ThermoFisher | D3922 |
| Complete-Mini Protease Inhibitor | Sigma-Aldrich | 11836170001 |
| DAPI | ThermoFisher | P36966 |
| DC Protein Assay kit | Biorad | 5000111 |
| DMEM | Sigma-Aldrich | D6546 |
| DNase | Qiagen | 79254 |
| FBS | Gibco | 10270-106 |
| gDNA extraction kit | Qiagen | 13323 |
| GlutaMax | ThermoFisher | 35050038 |
| Immobilon Western Chemiluminescent HRP Substrate | Millipore | WBKLS0500 |
| Isopropanol | Honeywell | 33539 |
| L-glutamine | Sigma-Aldrich | G7513 |
| LipidTOX DeepRed | ThermoFisher | H34477 |
| Lipofectamine 3000 | ThermoFisher | L3000008 |
| Lipofectamine RNAiMax | ThermoFisher | 13778150 |
| MEM Non-essential Amino Acid | Sigma-Aldrich | M7145 |
| Mfn1 siRNA-1 | ThermoFisher | s85004 |
| Mfn1 siRNA-2 | ThermoFisher | s85002 |
| Mfn1 siRNA-3 | ThermoFisher | s85003 |
| Mfn2 siRNA pool | Dharmacon | L-046303-00-0005 |
| Mitotracker Orange CMTMRos | ThermoFisher | M7510 |
| NuPAGE 4x LDS buffer | ThermoFisher | NP0007 |
| NuPAGE MES runing buffer | ThermoFisher | NP0002 |
| Oil red O | Sigma-Aldrich | O0625 |
| OptiMEM | ThermoFisher | 31985062 |
| Penicillin-Streptomycin | Sigma-Aldrich | P0781 |
| PhosSTOP Phosphatase Inhibitor | Roche | 4906837001 |
| Polybrene | EMD Millipore | TR-1003-G |
| Protein ladder | Biorad | 161-0374 |
| RIPA Buffer | SERVA Electrophoresis | 39244.01 |
| RNeasy Mini RNA extraction kit | Qiagen | 74106 |
| Sodium pyruvate | Sigma-Aldrich | S8636 |
| Trypsin-EDTA | Sigma-Aldrich | T4674 |
| Tween 20 | Sigma-Aldrich | P1379 |
| **Cell line** | **Supplier** | **CatID** |
| 3T3-L1 | D. Fazakerly (IMS, Uni Cambridge) | |
| Mfn1^-/-^ MEFs | ATCC | CRL-2992 |
| Mfn1^-/-^2^-/-^ MEFs | ATCC | CRL-2994 |
| Mfn2^-/-^ MEFs | ATCC | CRL-2993 |
| Opa1^-/-^ MEFs | ATCC | CRL-2995 |
| Phoenix-AMPHO cells | ATCC | CRL-3213 |
| WT MEFs | ATCC | CRL-2991 |

**Supplementary Table 1**: **Reagents and cell lines.** List of all reagents and equipment used in this study.
