# Supplementary material for: Loss of Mfn1 but not Mfn2 enhances adipogenesis
Source: PLoS One. 2024 Dec 31;19(12):e0306243. doi: 10.1371/journal.pone.0306243 (PMC11687706; doi:10.1371/journal.pone.0306243)
Supplement: S2 Table — List of all primary and secondary antibodies used in this study. (DOCX) [file pone.0306243.s009.docx]

| **Primary** | **Supplier** | **CatID** | **Concentration** | **Diluent** | **2ary** |
| --- | --- | --- | --- | --- | --- |
| Anti-mouse HRP | Cell signalling | 70765 | 1 in 5000 | 5% milk | N/A |
| Anti-rabbit HRP | Cell signalling | 70751 | 1 in 5000 | 5% milk | N/A |
| Adipoq | Abcam | ab85827 | 1 in 1000 | 5% milk | Rabbit |
| Akt (total) | Cell signalling | 4691 | 1 in 1000 | 5% milk | Rabbit |
| Phospho-Akt (Ser473) | Cell signalling | 9271 | 1 in 1000 | 5% milk | Rabbit |
| Beta-tubulin | Abcam | ab6046 | 1 in 1000 | 5% milk | Rabbit |
| Calnexin | Abcam | ab22595 | 1 in 5000 | 5% BSA | Rabbit |
| Drp1 | Abcam | ab56788 | 1 in 1000 | 5% milk | Rabbit |
| Fabp4 | Cell signalling | 2120 | 1 in 1000 | 5% milk | Rabbit |
| Fis1 | Proteintech | 10956-1-AP | 1 in 2000 | 5% milk | Rabbit |
| Gapdh | GeneTex | GTX100118 | 1 in 5000 | 5% milk | Rabbit |
| Glut1 | Cell signalling | 12939 | 1 in 1000 | 5% milk | Rabbit |
| Glut4 | Boster | PA1722 | 1 in 1000 | 5% milk | Rabbit |
| Insr | Santa cruz | sc-57342 | 1 in 1000 | 5% milk | Mouse |
| Mfn1 | Abcam | Ab126575 | 1 in 250 | 5% milk | Mouse |
| Mfn2 | Cell signalling | D2D10 | 1 in 1000 | 5% milk | Rabbit |
| Opa1 | BD Biosciences | 612606 | 1 in 1000 | 5% milk | Mouse |
| OXPHOS cocktail | Abcam | Ab110413 | 1 in 1000 | 5% milk | Mouse |
| Plin1 | Progen | GP29 | 1 in 1000 | 5% BSA | Guinea pig |
| PPAR-gamma | Cell signalling | 81B8 | 1 in 1000 | 5% BSA | Rabbit |
| Tom20 [immunoblots] | Abcam | Ab56783 | 1 in 500 | 5% milk | Mouse |
| Tom20 [immunofluorescence] | Proteintech | 11802-1-AP | 1 in 1000 | 5% BSA | Alexa Fluor 488 |

**Supplementary Table 2**: **Antibodies used.** List of all primary and secondary antibodies used in this study.
