# Supplementary material for: Loss of Mfn1 but not Mfn2 enhances adipogenesis
Source: PLoS One. 2024 Dec 31;19(12):e0306243. doi: 10.1371/journal.pone.0306243 (PMC11687706; doi:10.1371/journal.pone.0306243)

Figure 1A

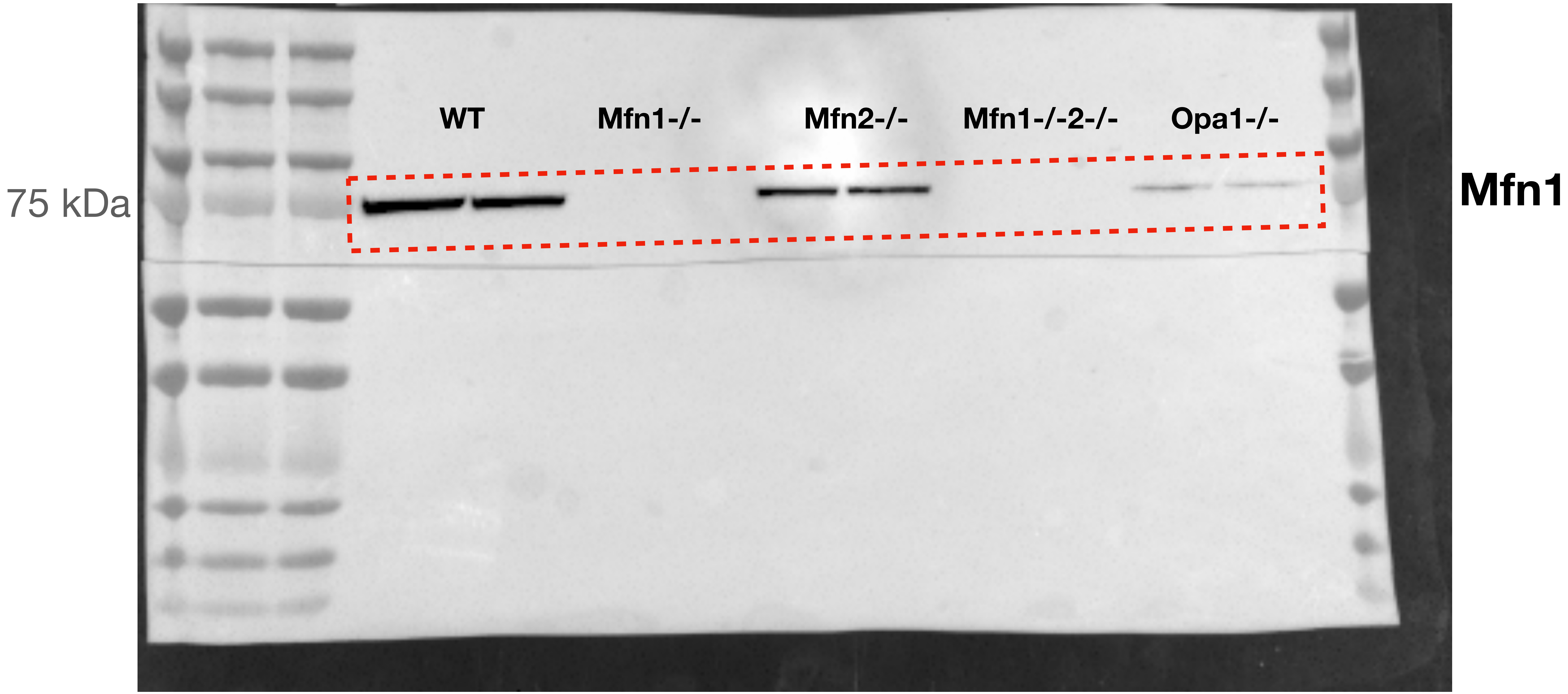

Figure 1A

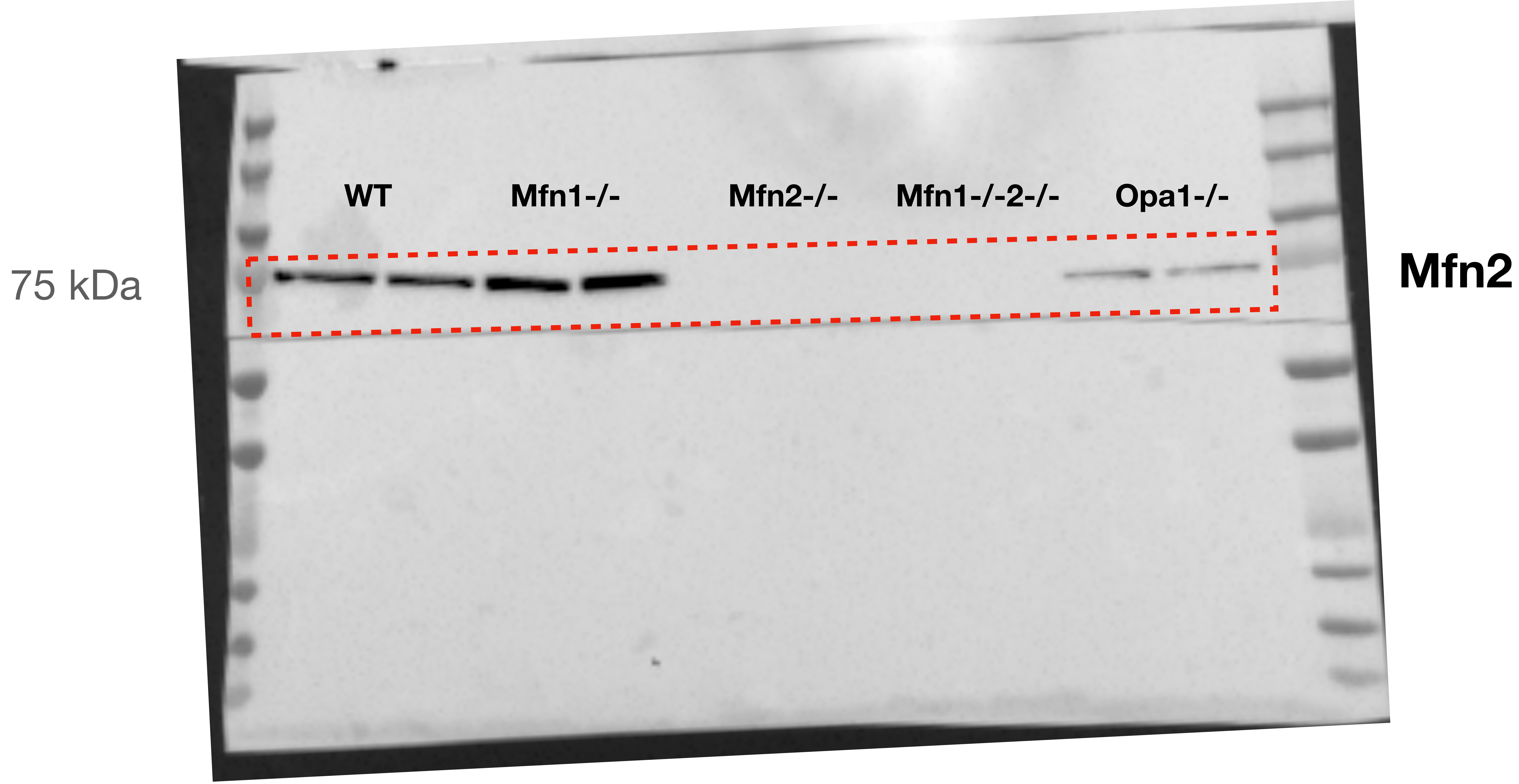

Figure 1A

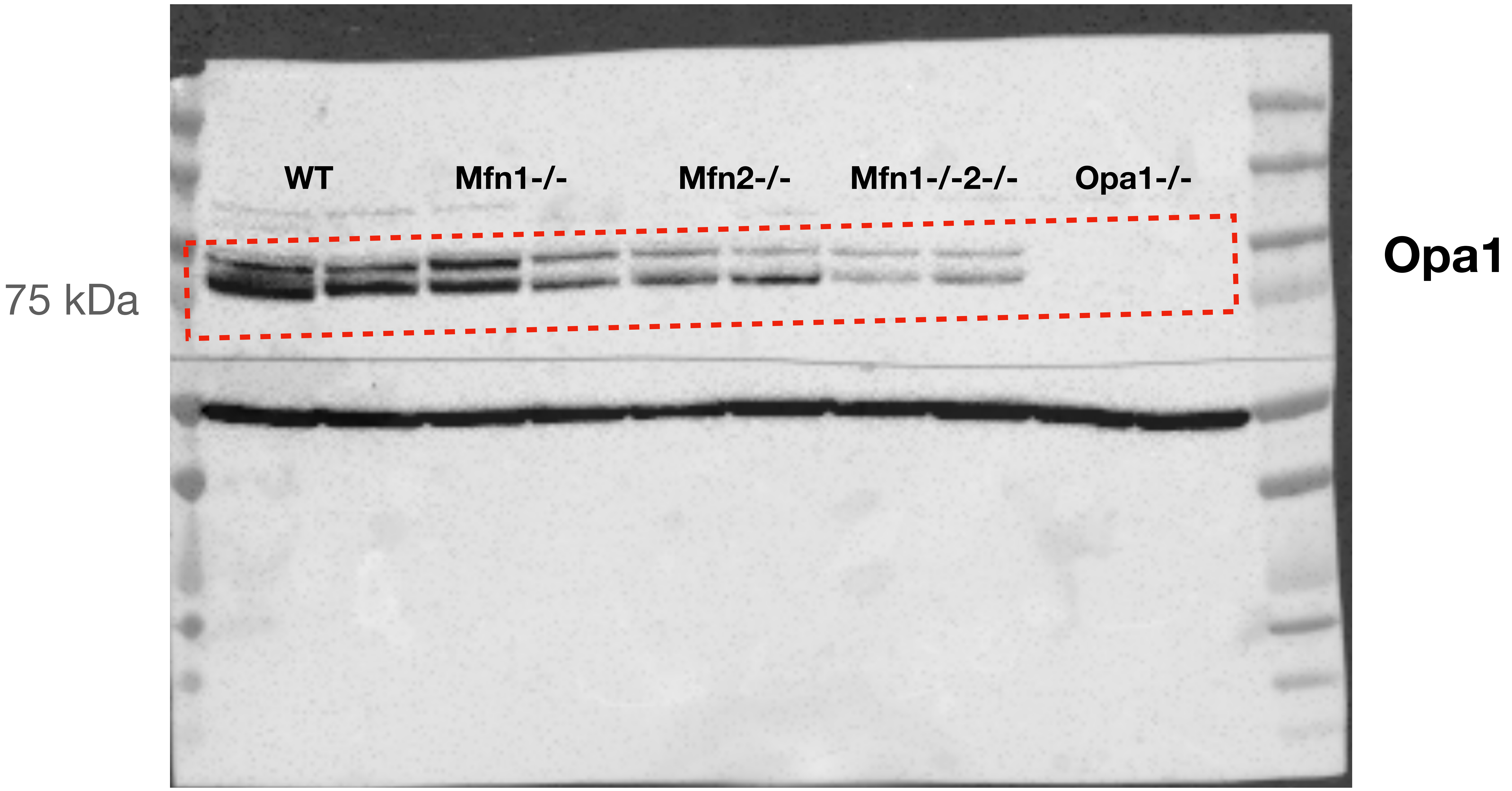

Figure 1A

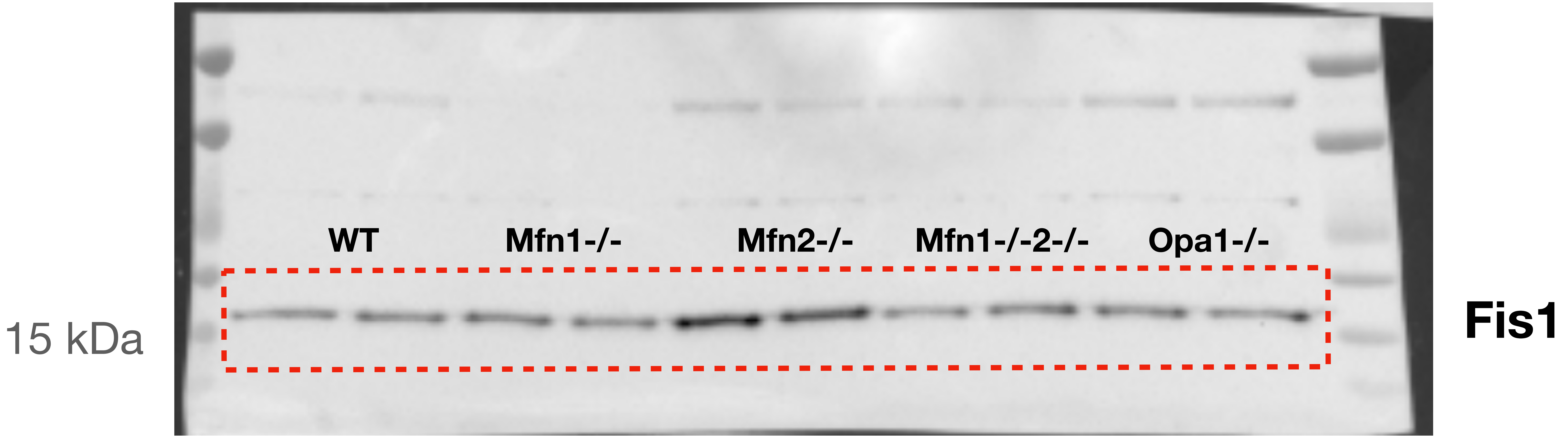

Figure 1A

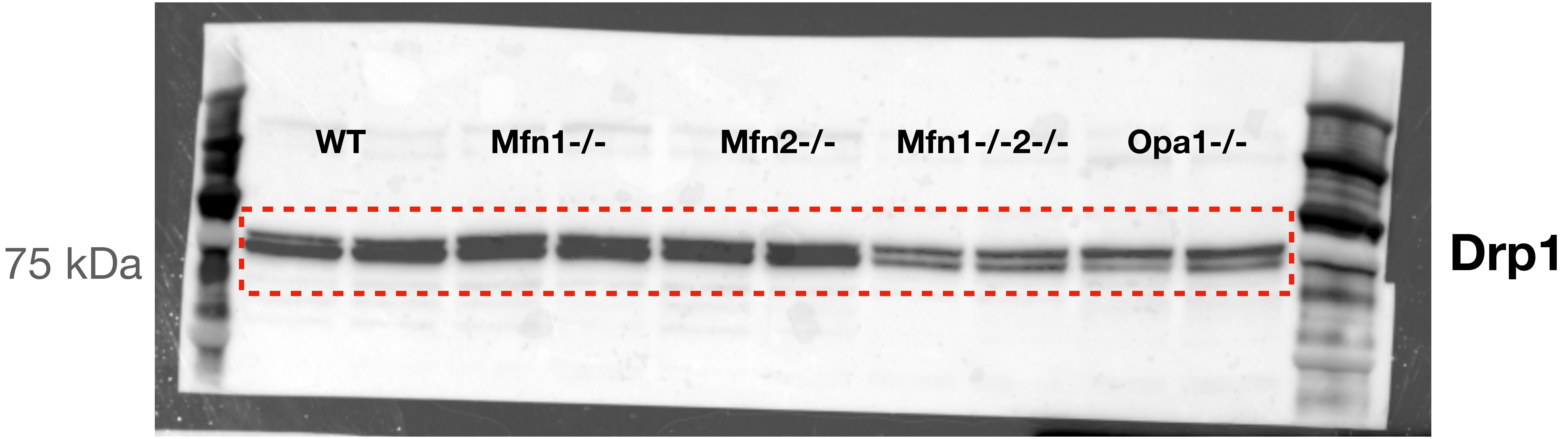

Figure 1A

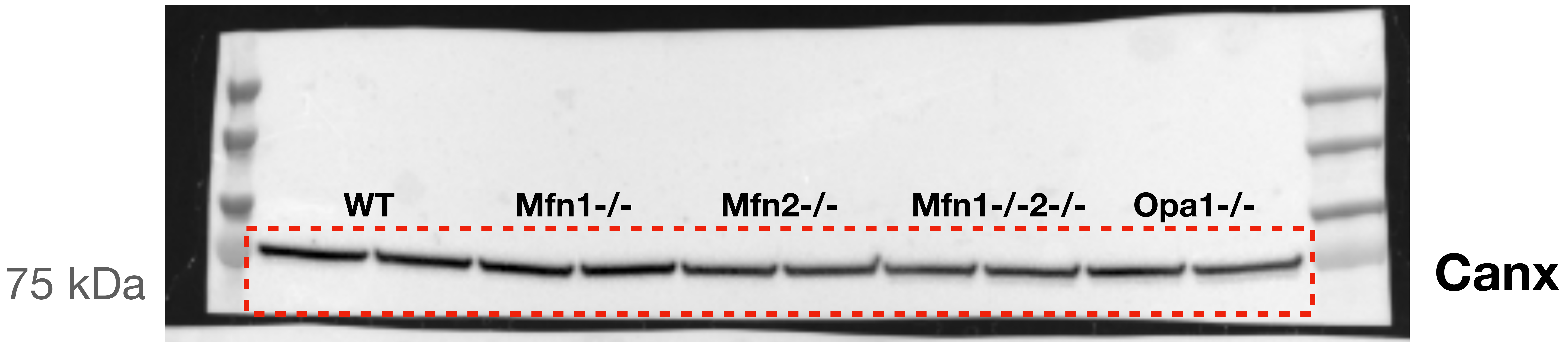

Figure 1F

Medium exposure

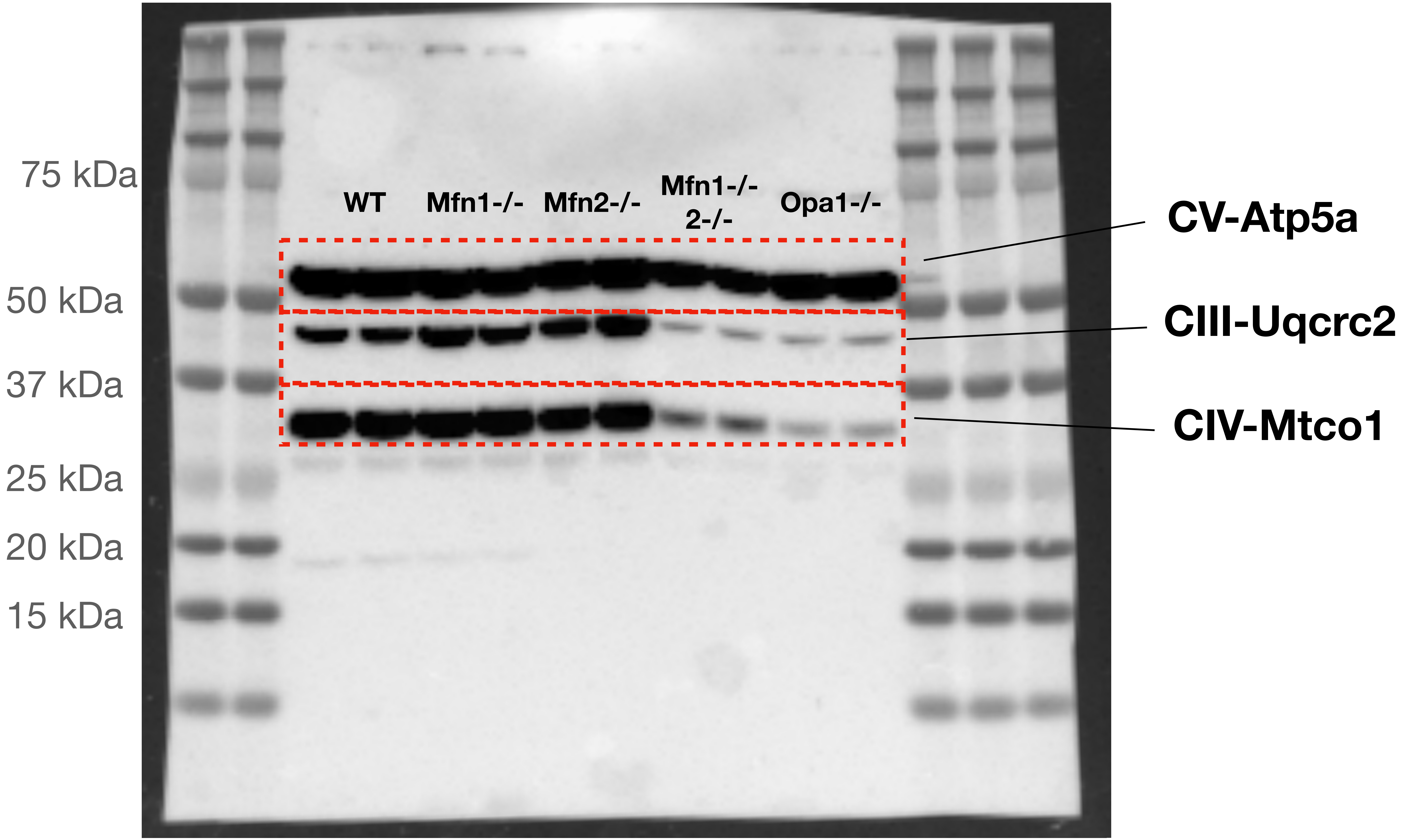

Figure 1F

High exposure

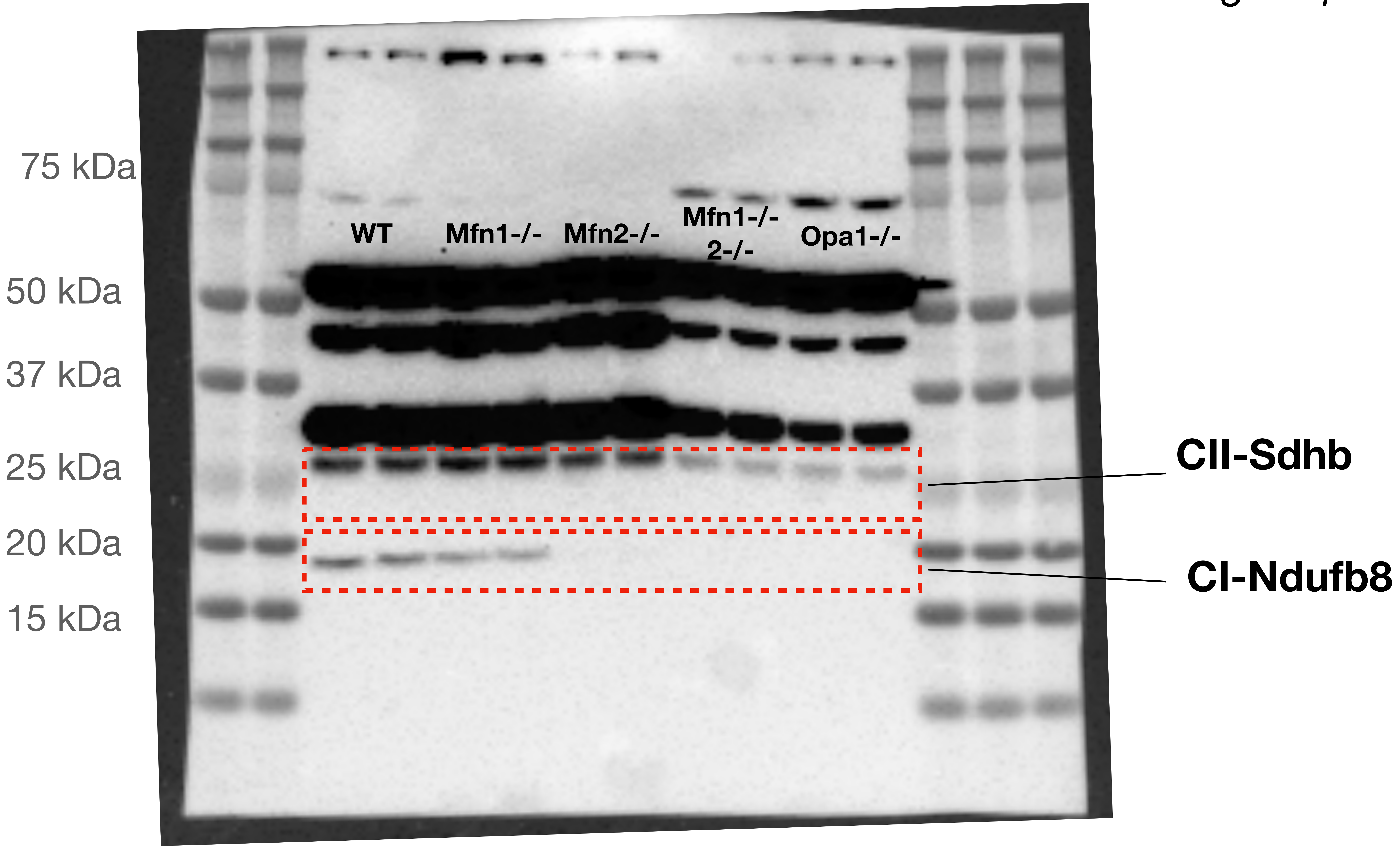

Figure 1F

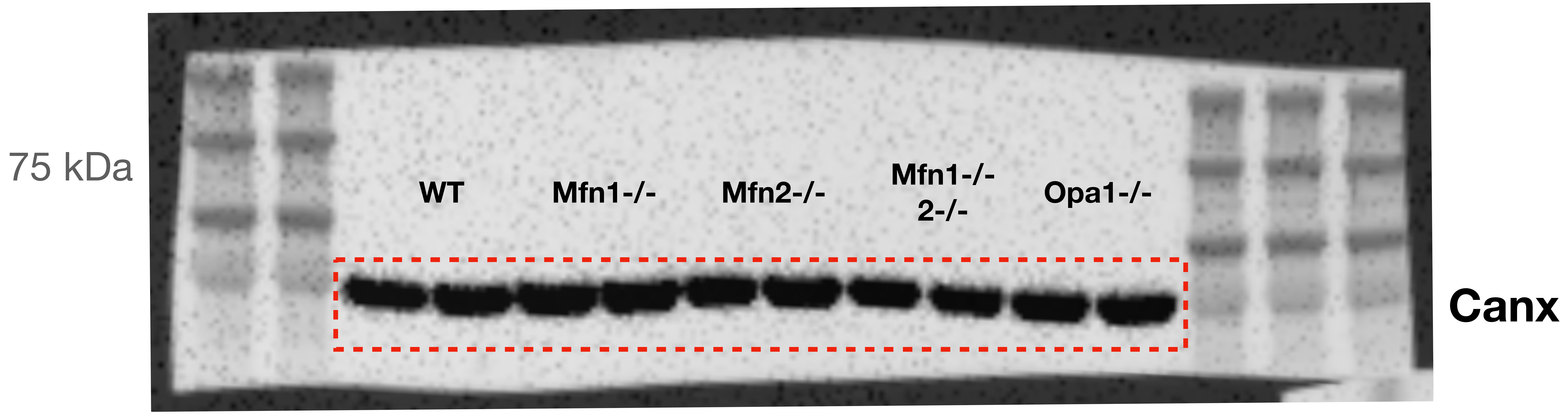

Figure 1F

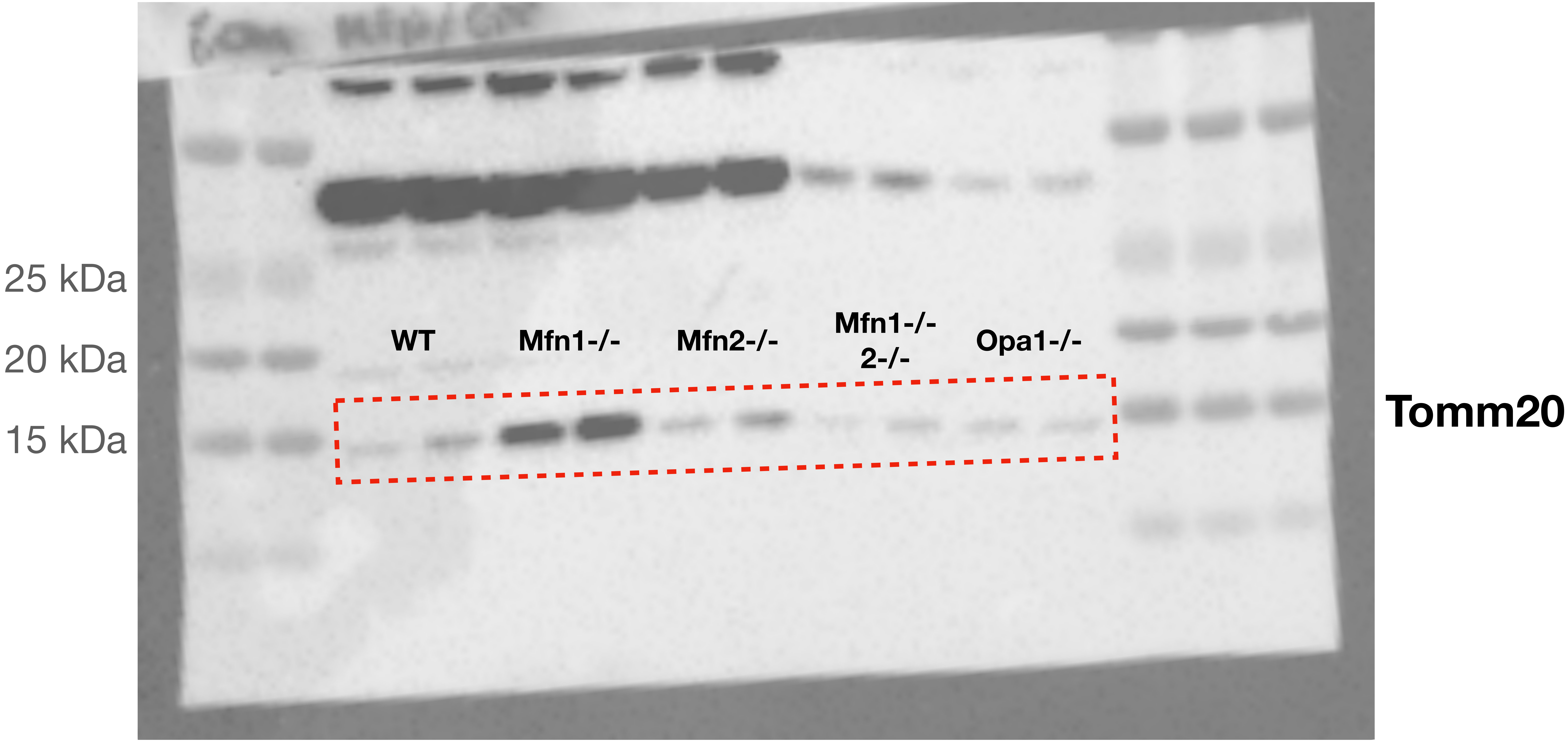

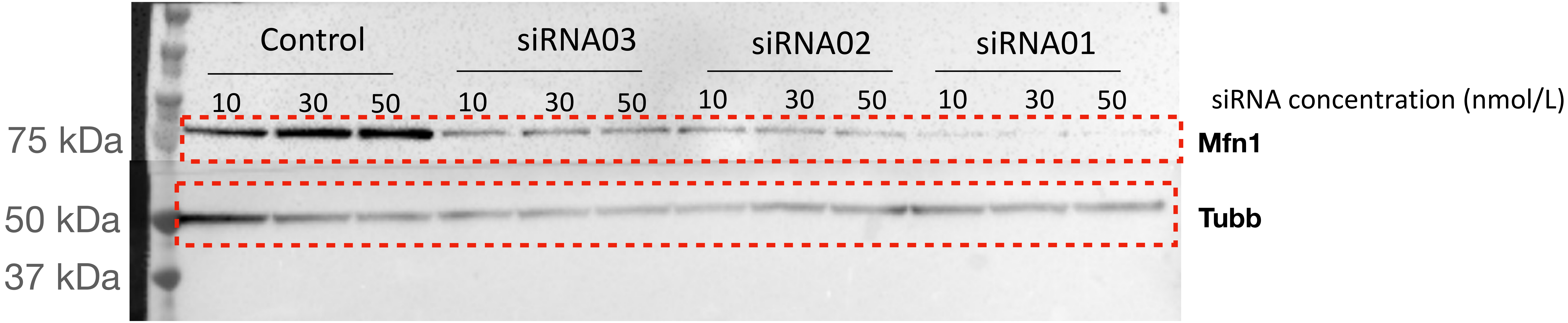
Control
siRNA03
siRNA02
siRNA01
10 30 50 10 30 50 10 30 50 10 30 50
siRNA concentration (nmol/L)
75 kDa
50 kDa
37 kDa
Mfn1
Tubb

Figure 2D

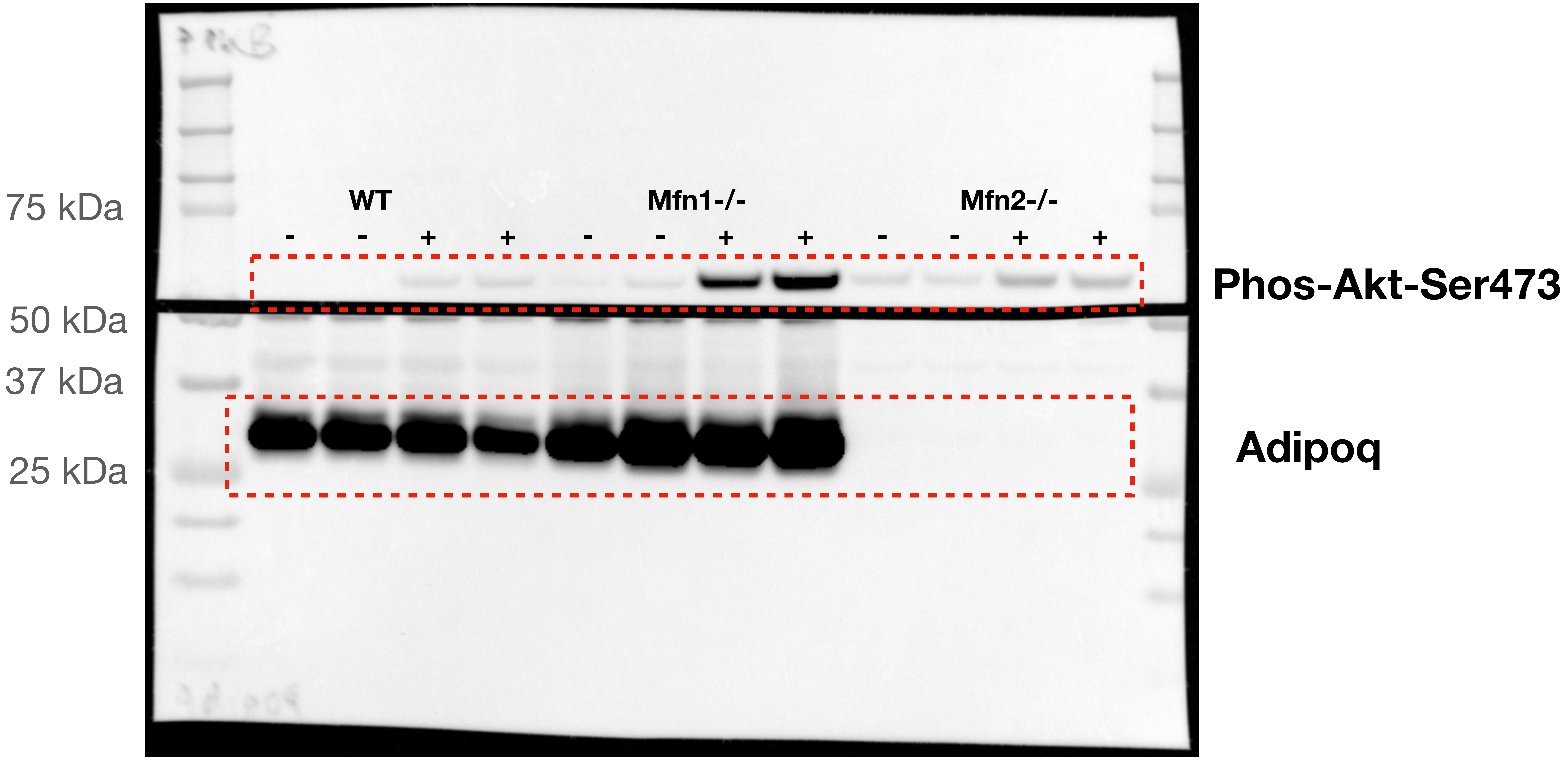

Figure 2D

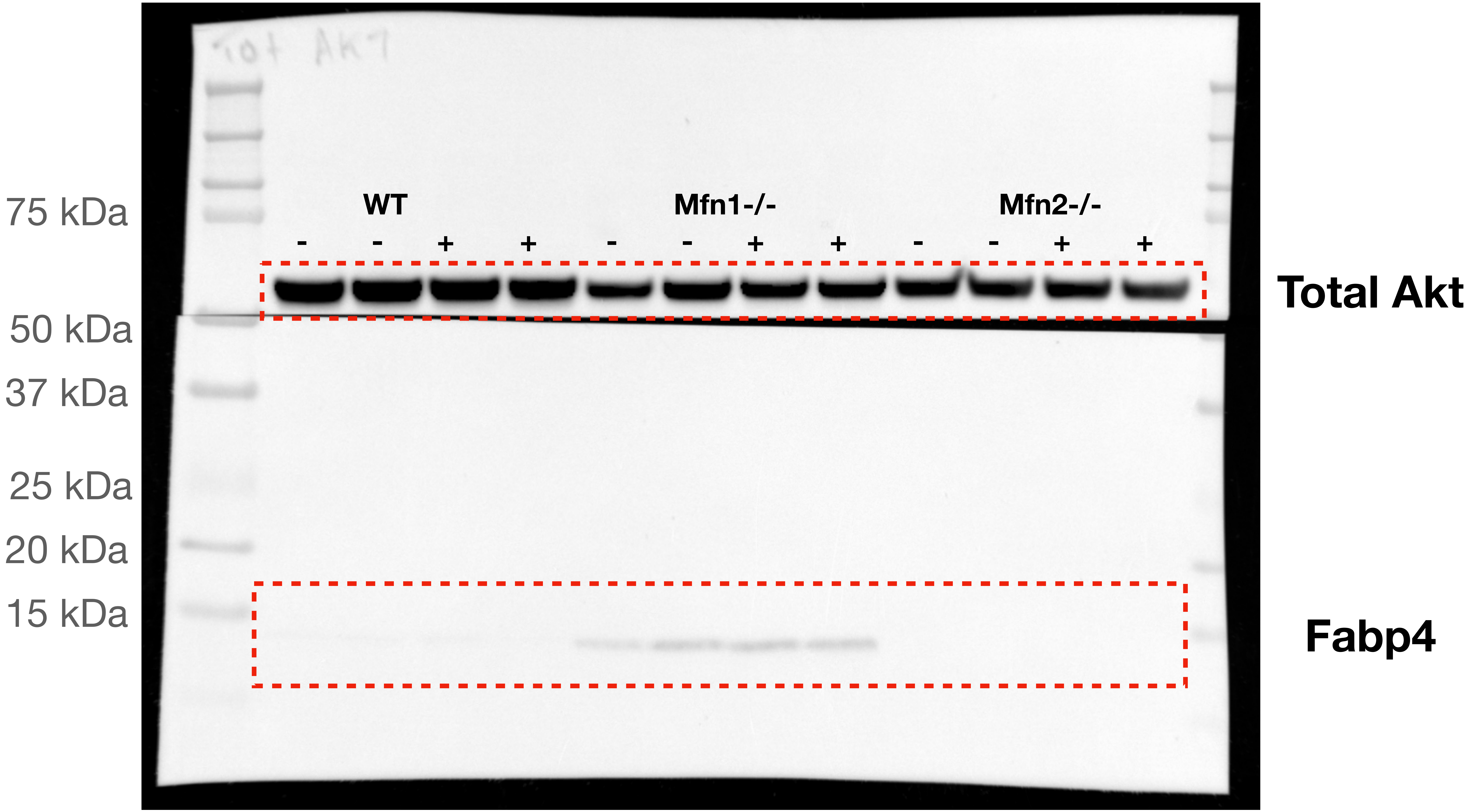

Tot AKT
75 kDa
50 kDa
37 kDa
25 kDa
20 kDa
15 kDa
WT
Mfn1-/-
Mfn2-/-
- - + + - - + + - - + +
Total Akt
Fabp4

Figure 2D

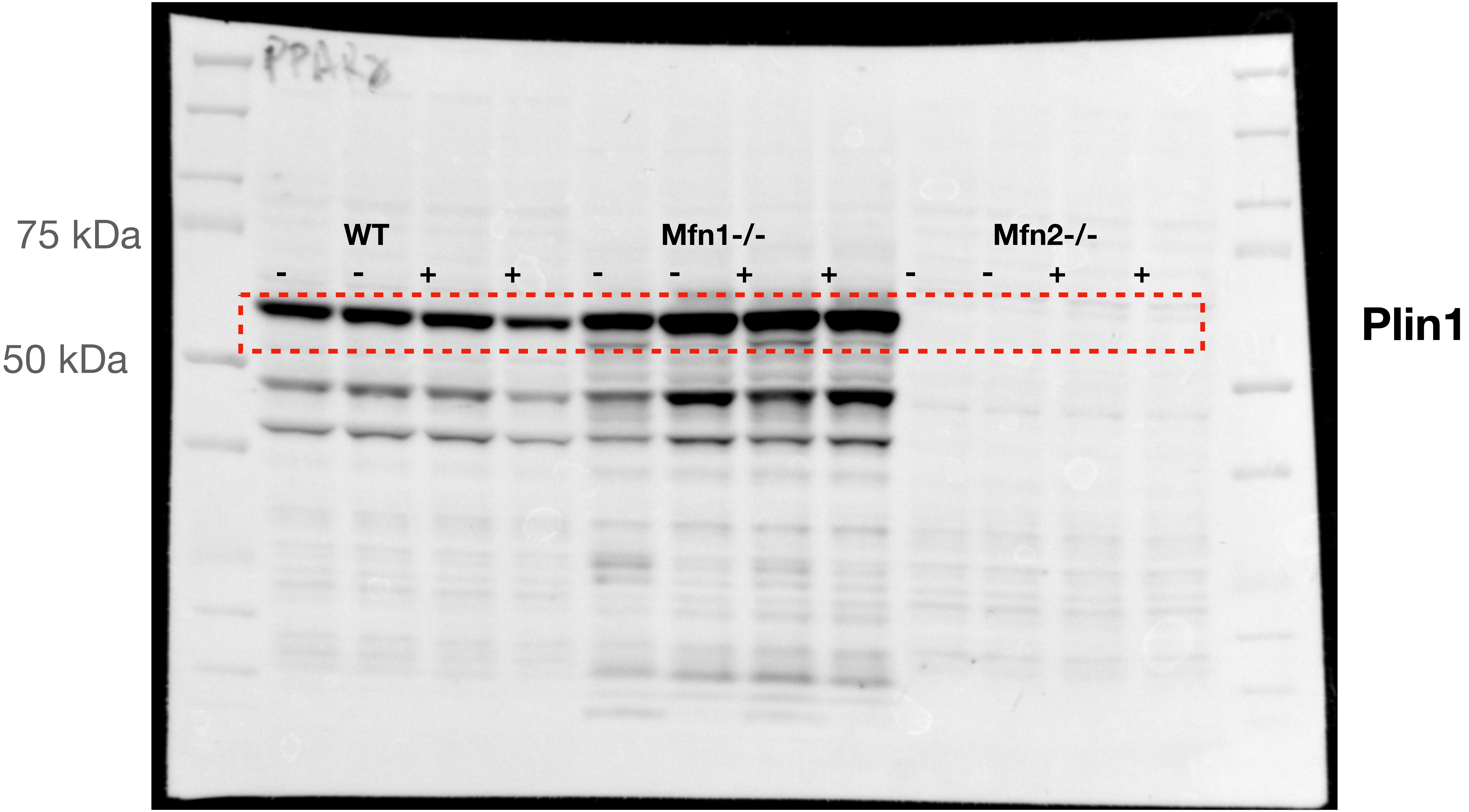

Figure 2D

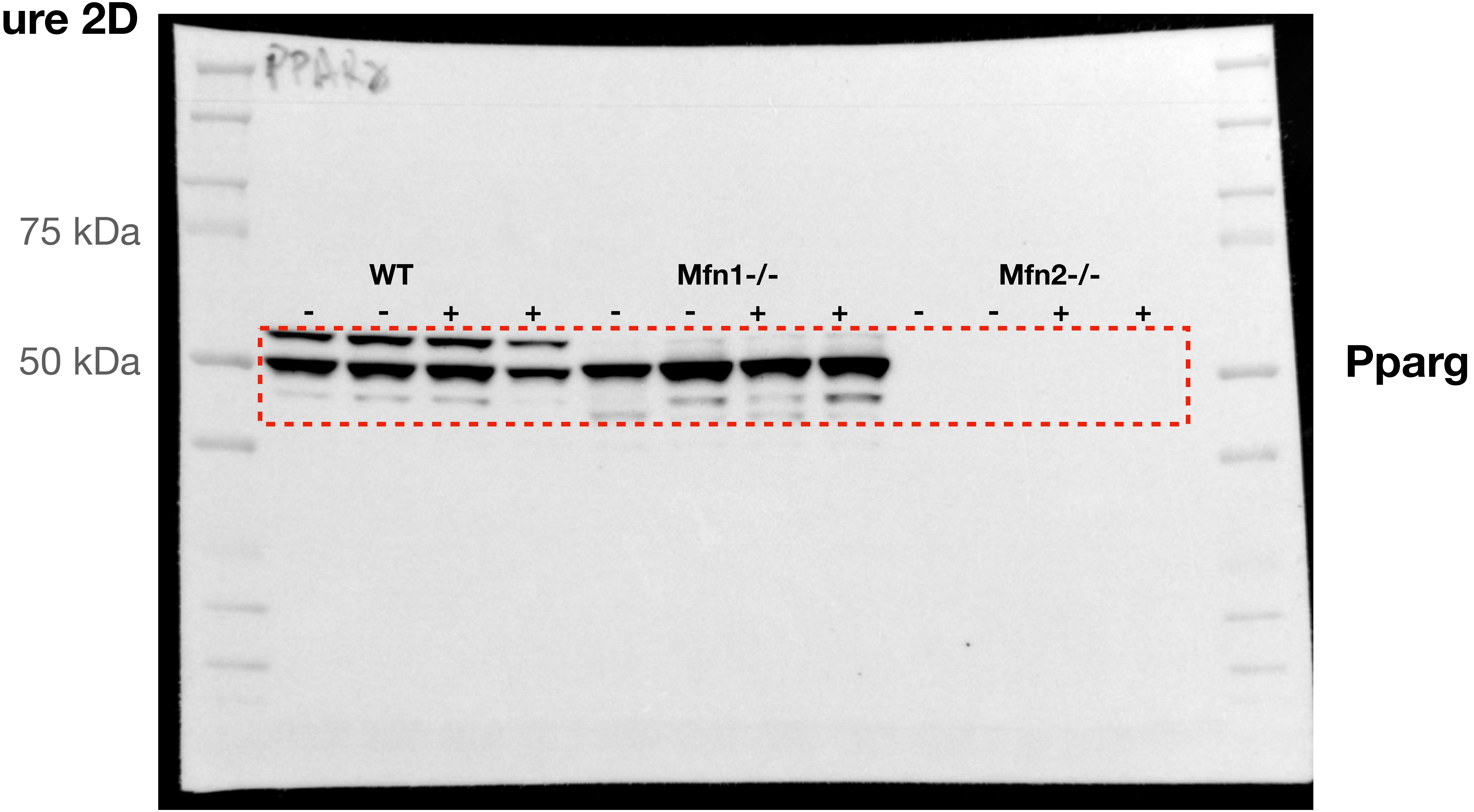

Figure 2D

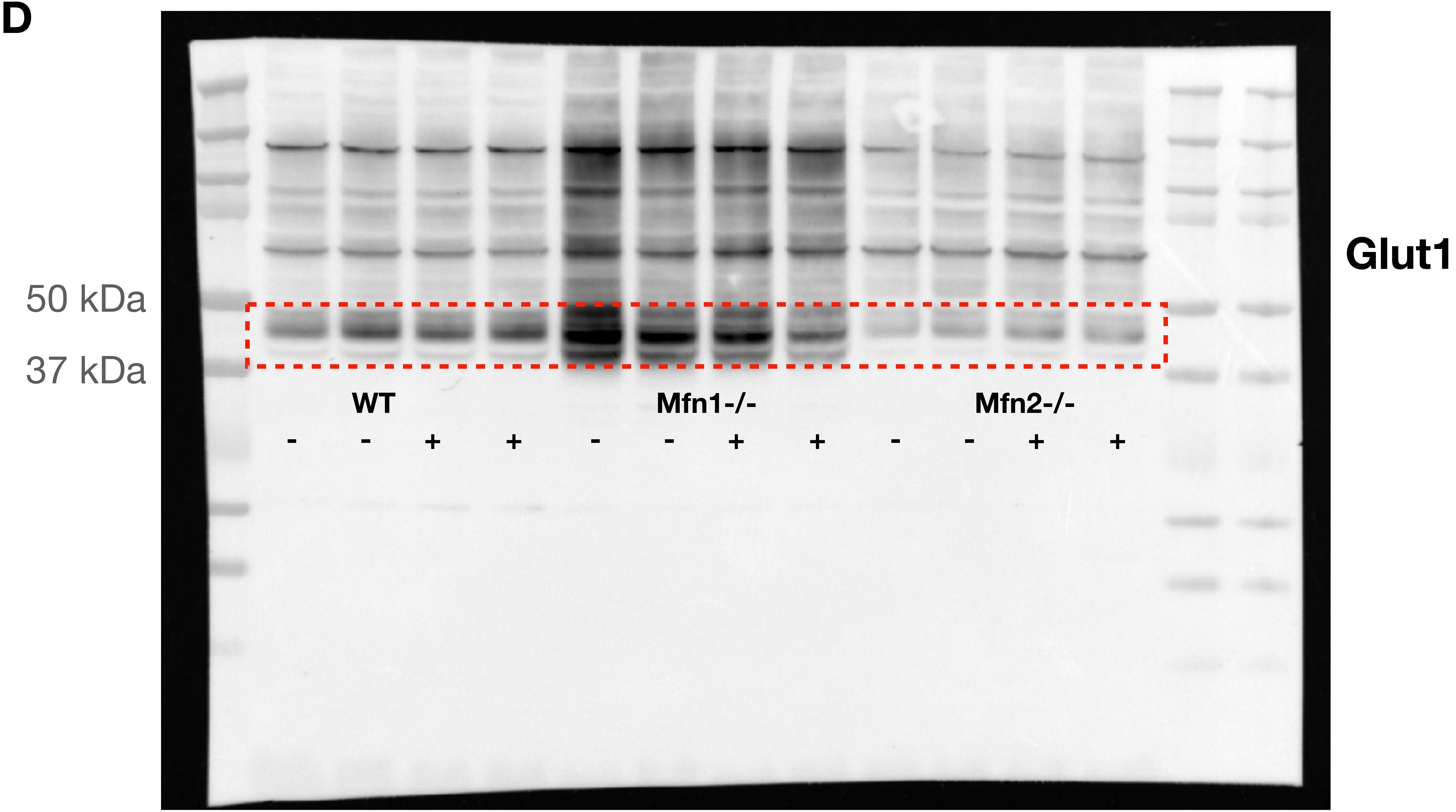

Figure 2D

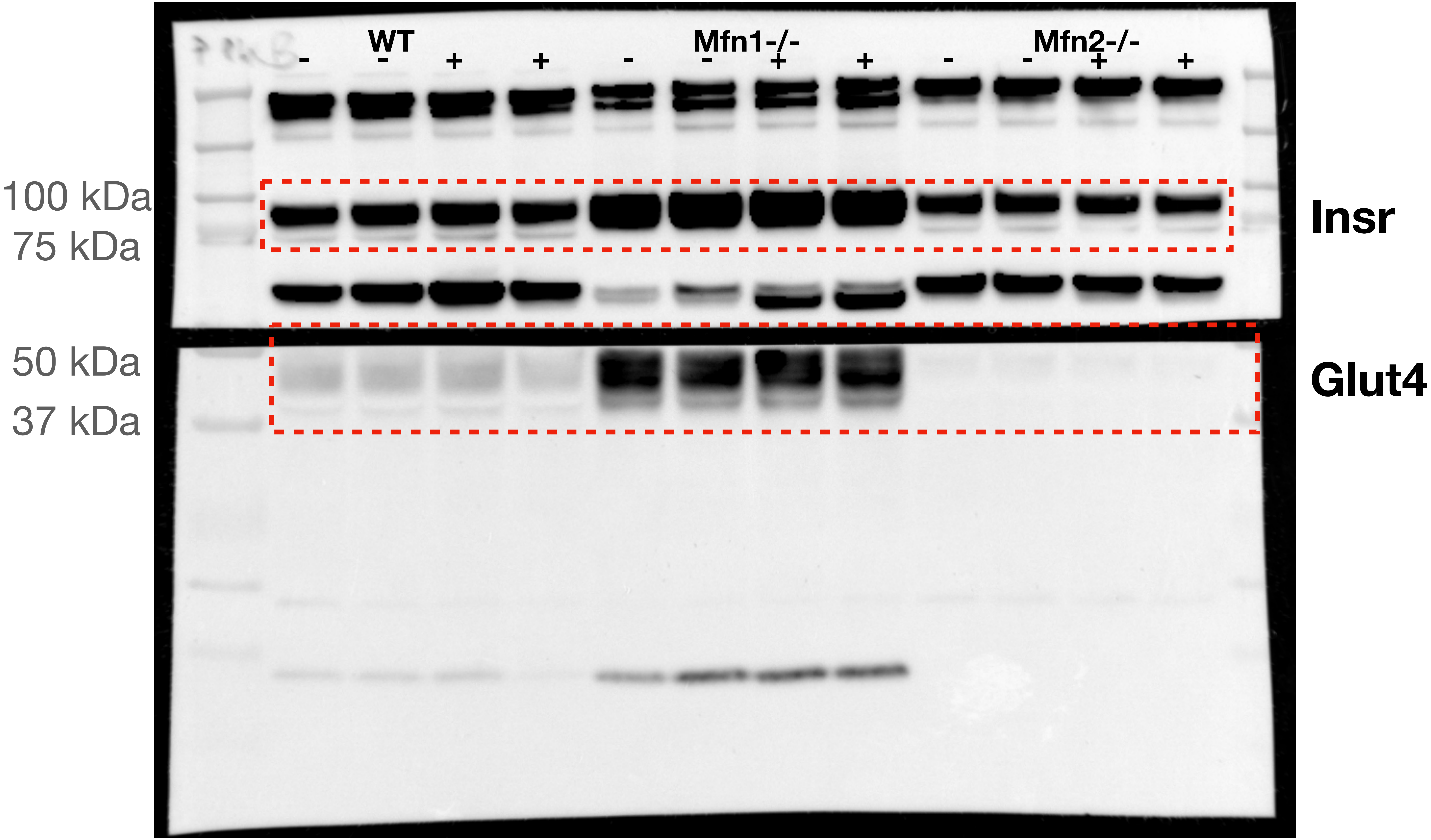

Figure 2D

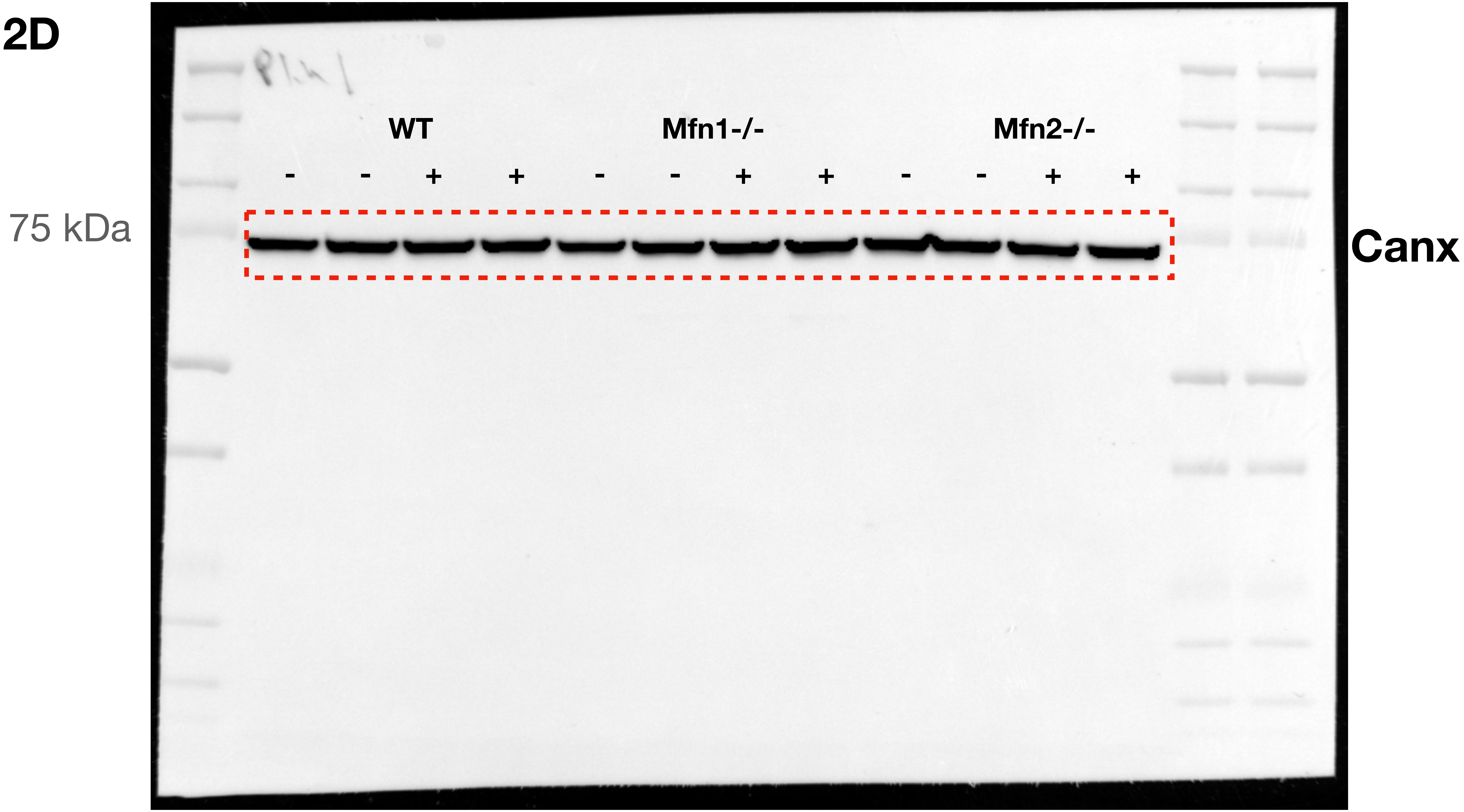

Figure 4A

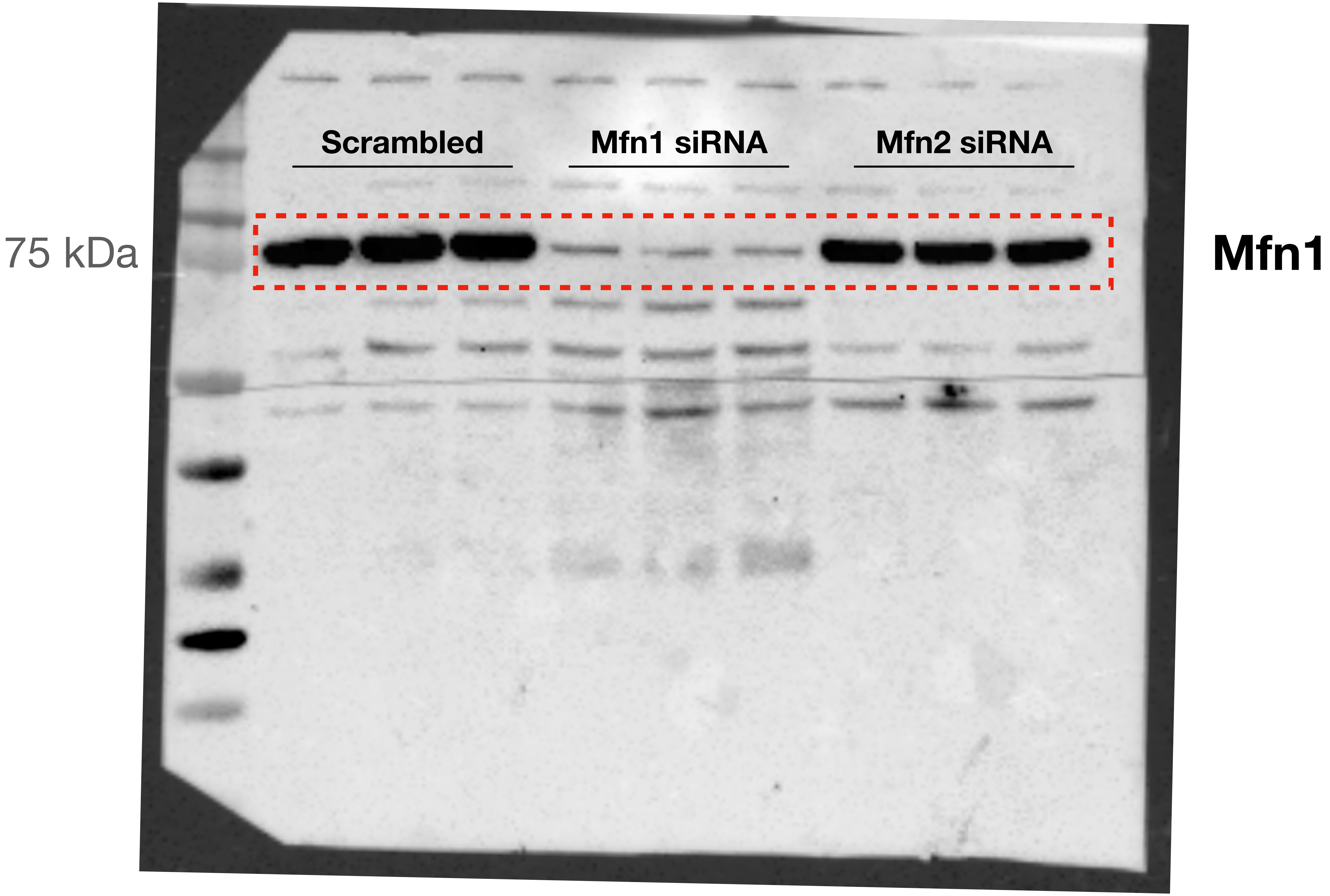

Figure 4A

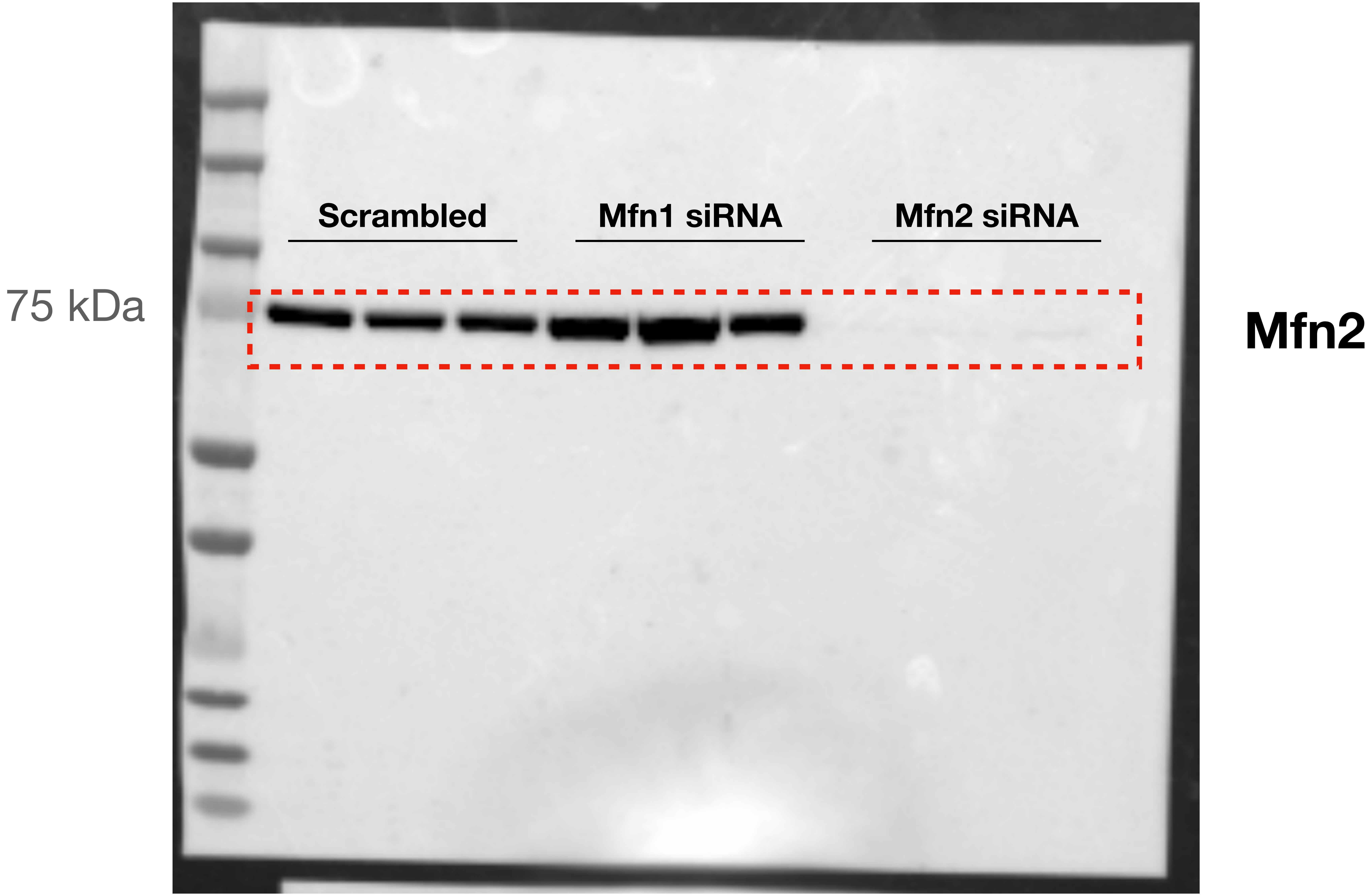

Figure 4A

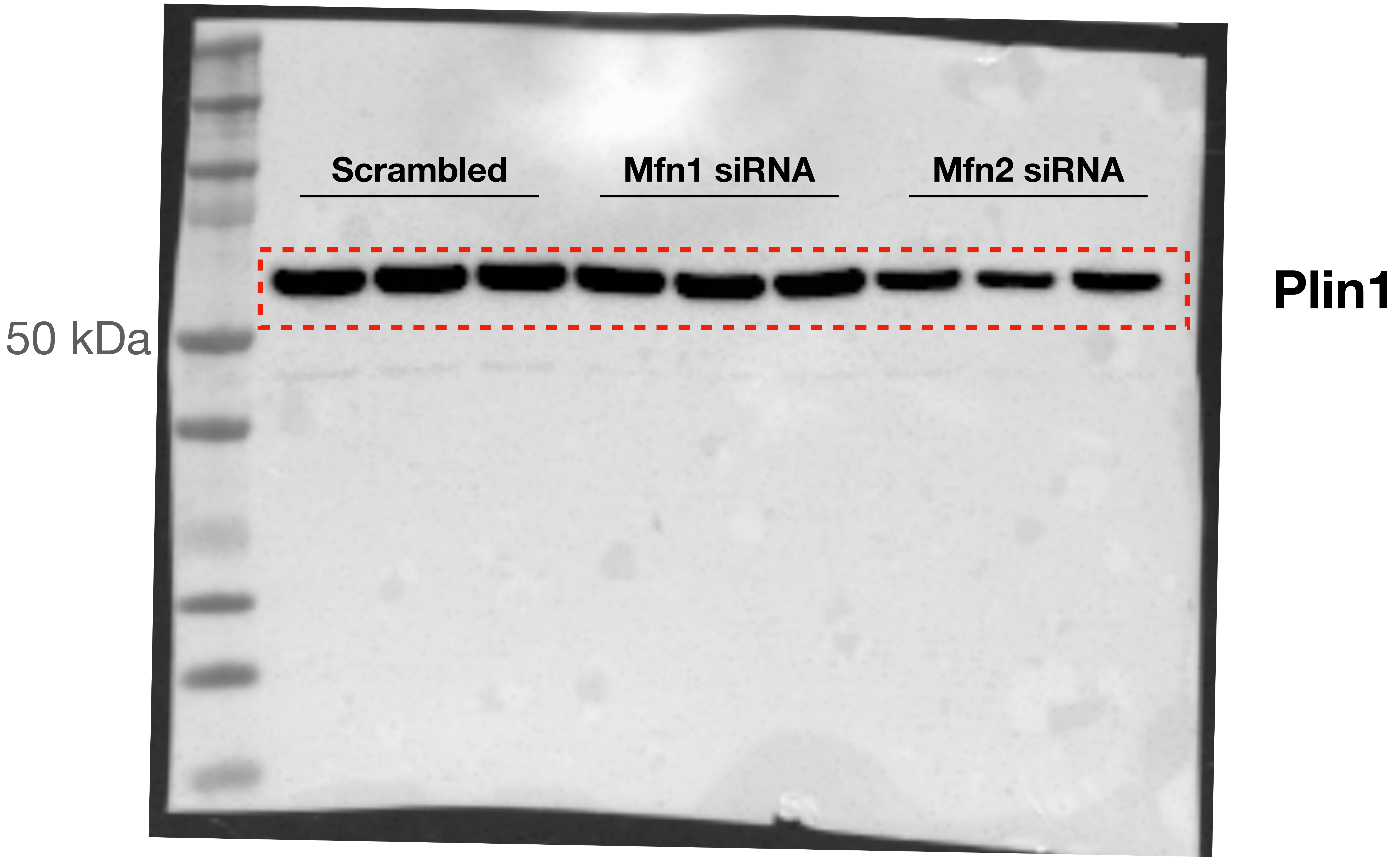

Figure 4A

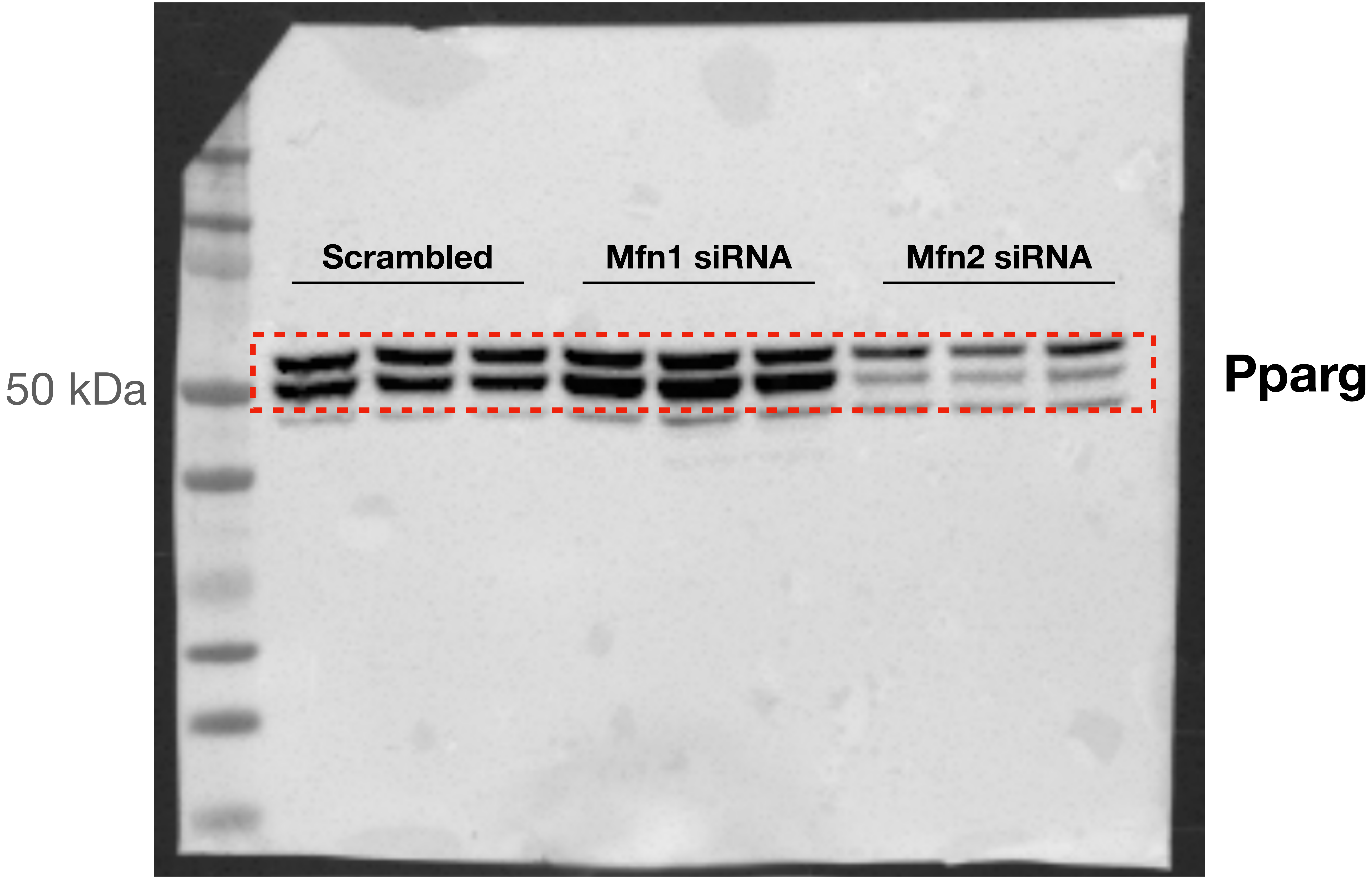

Figure 4A

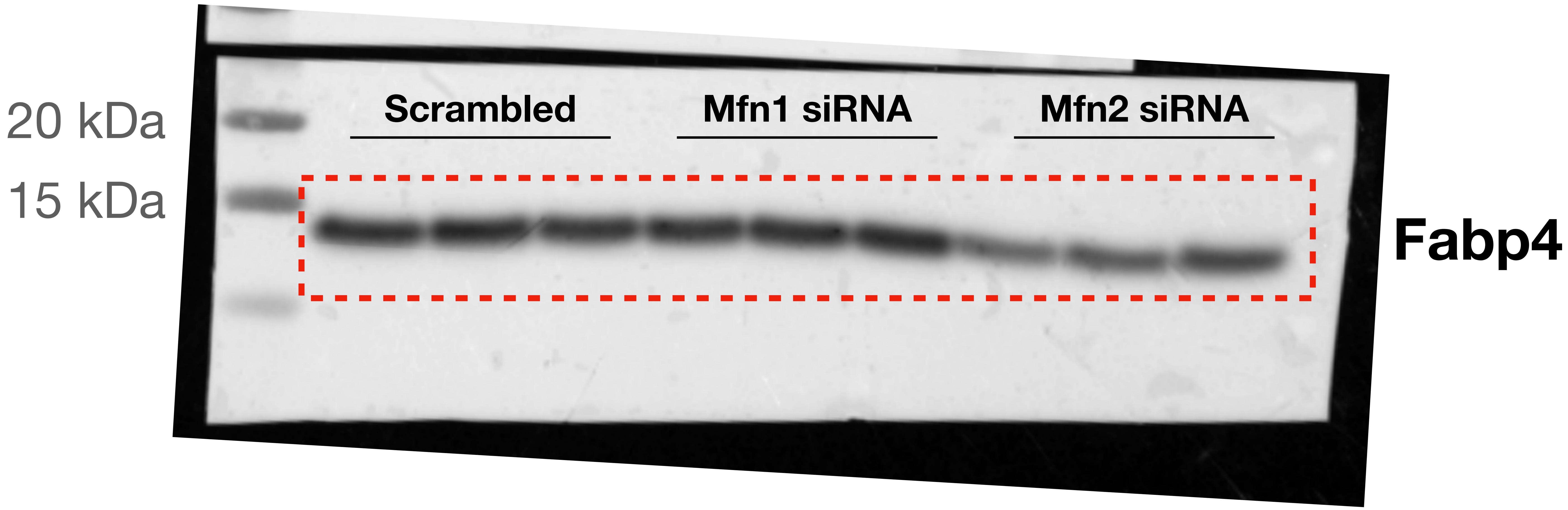

Figure 4A

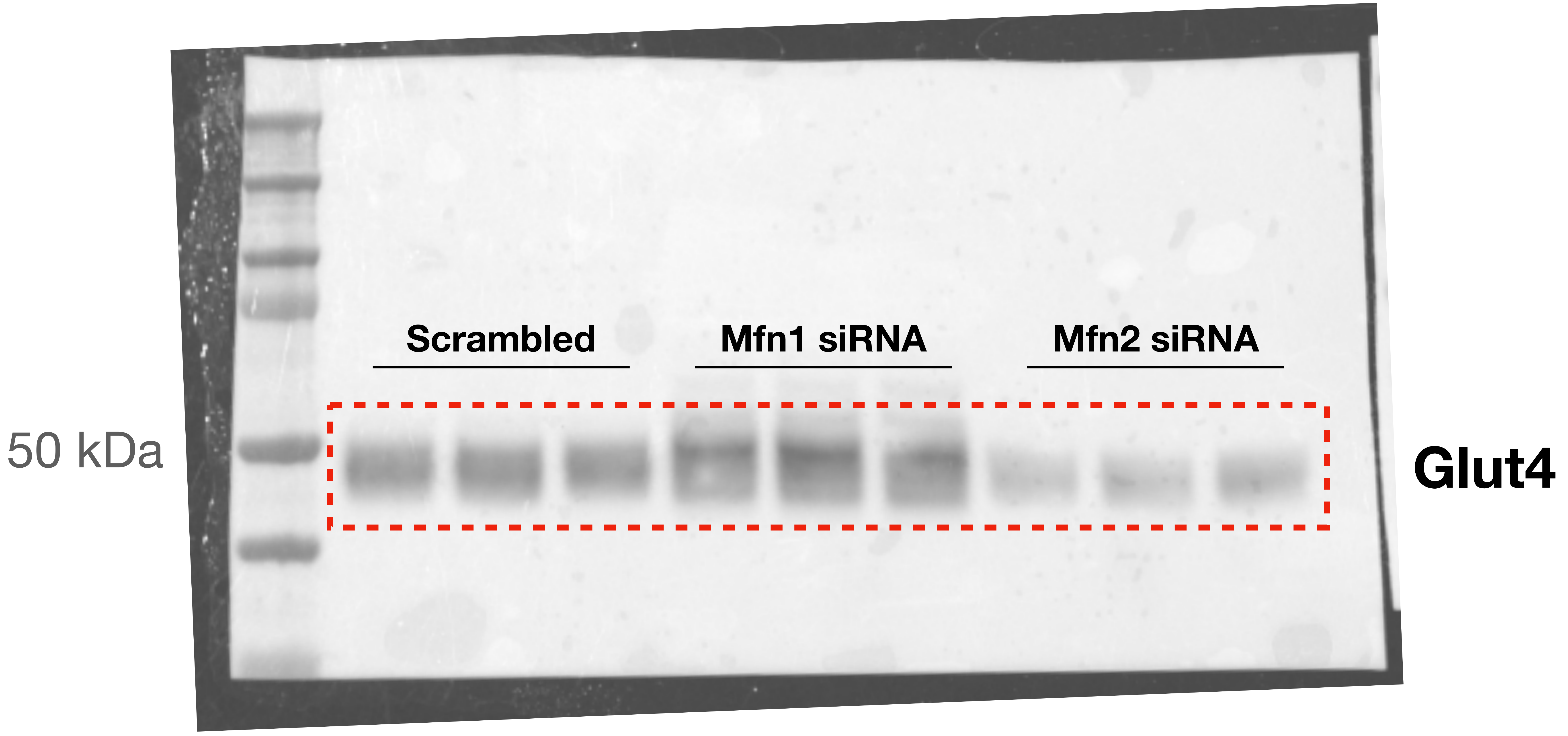

Figure 4A

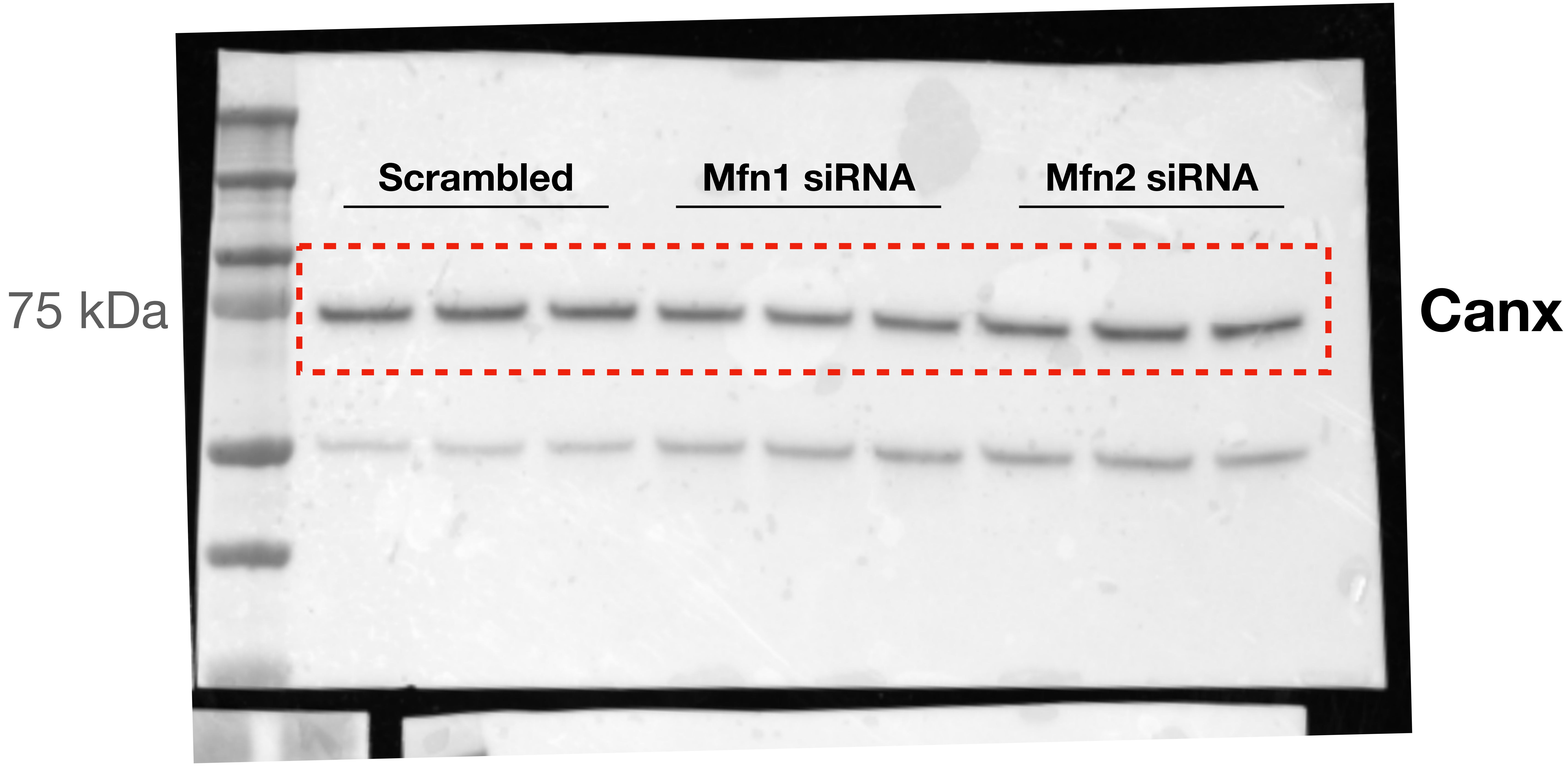

S3 Figure A

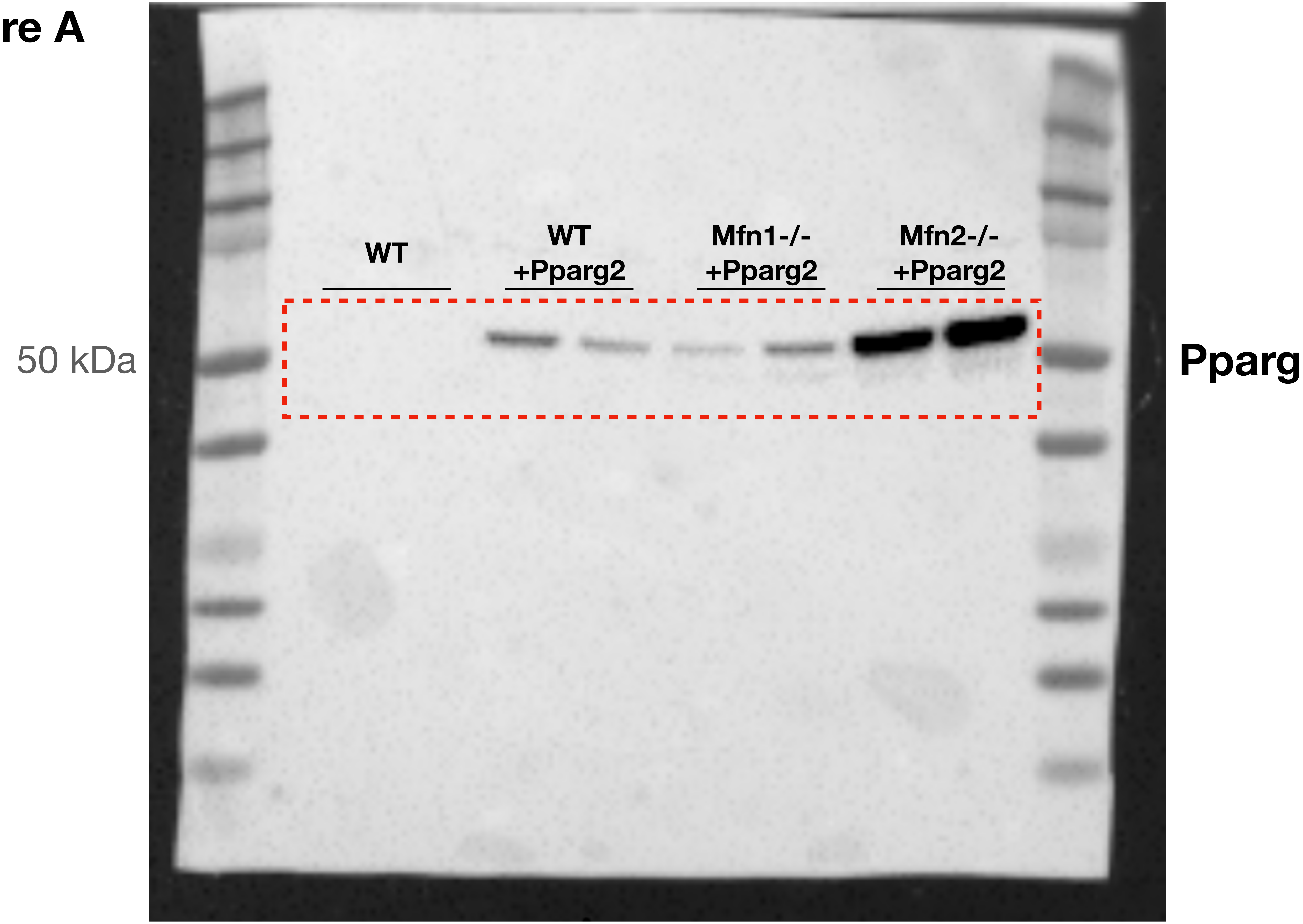

S3 Figure A

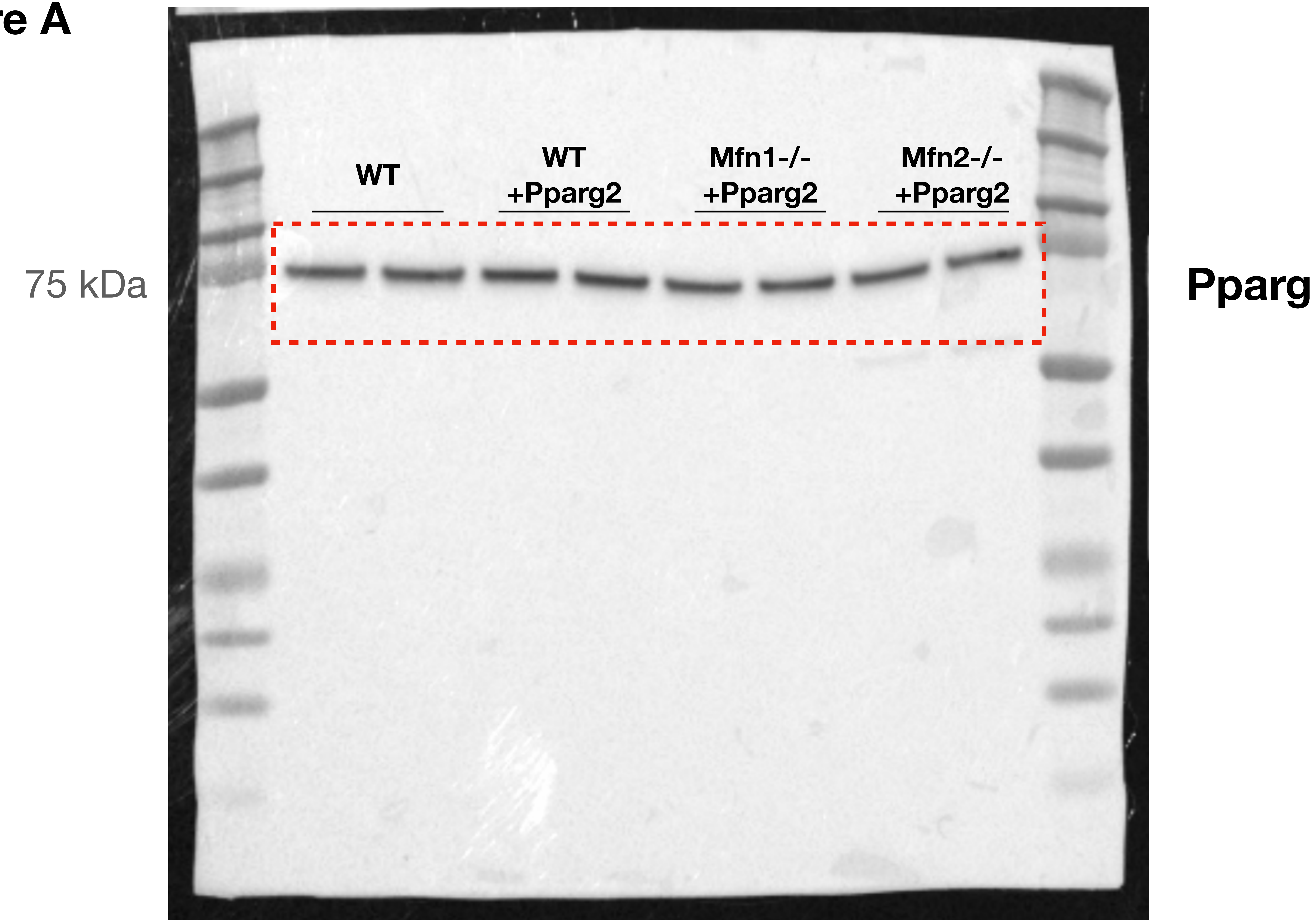

S3 Figure E

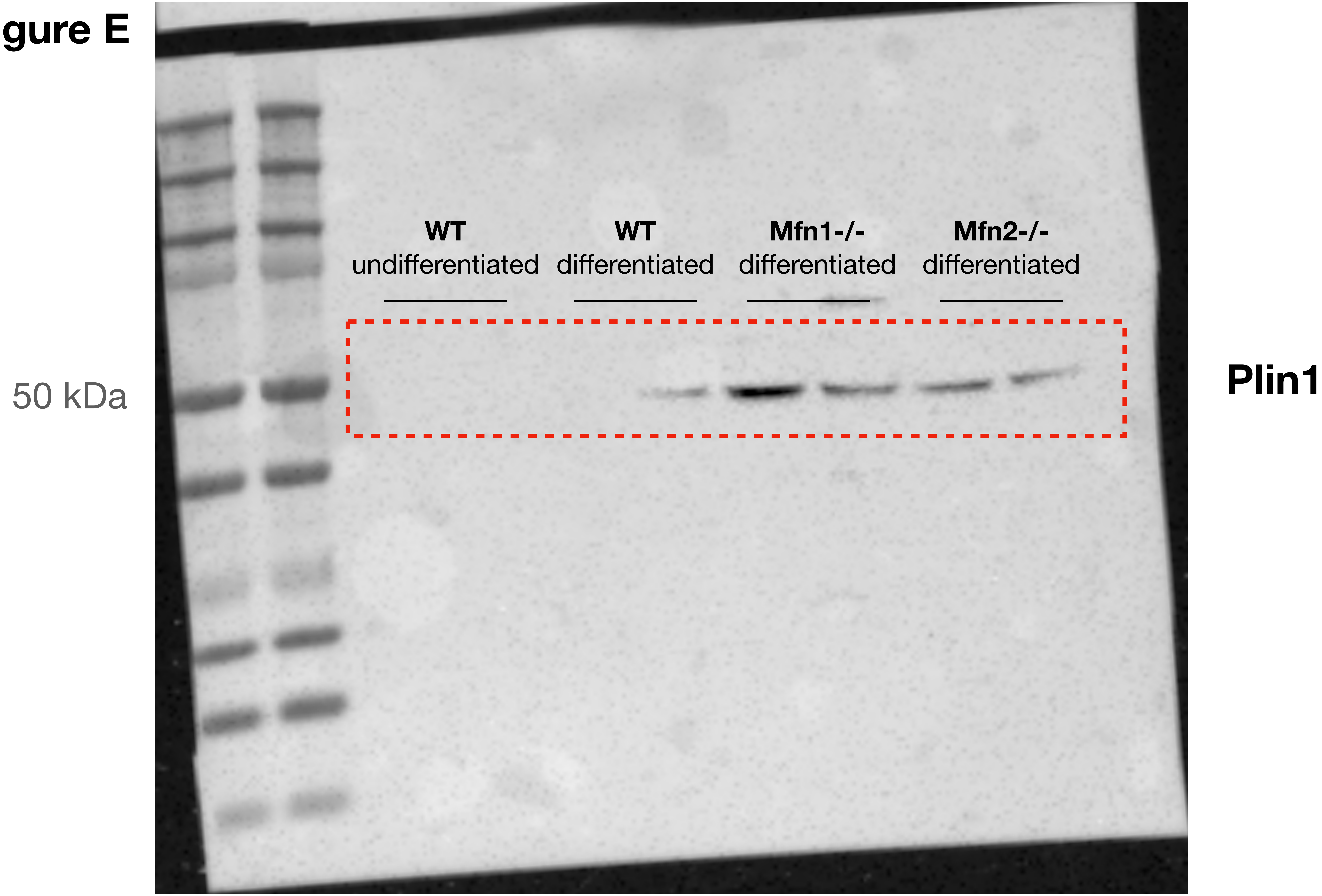
50 kDa
WT
undifferentiated
WT
differentiated
Mfn1-/-
differentiated
Mfn2-/-
differentiated
Plin1

S3 Figure E

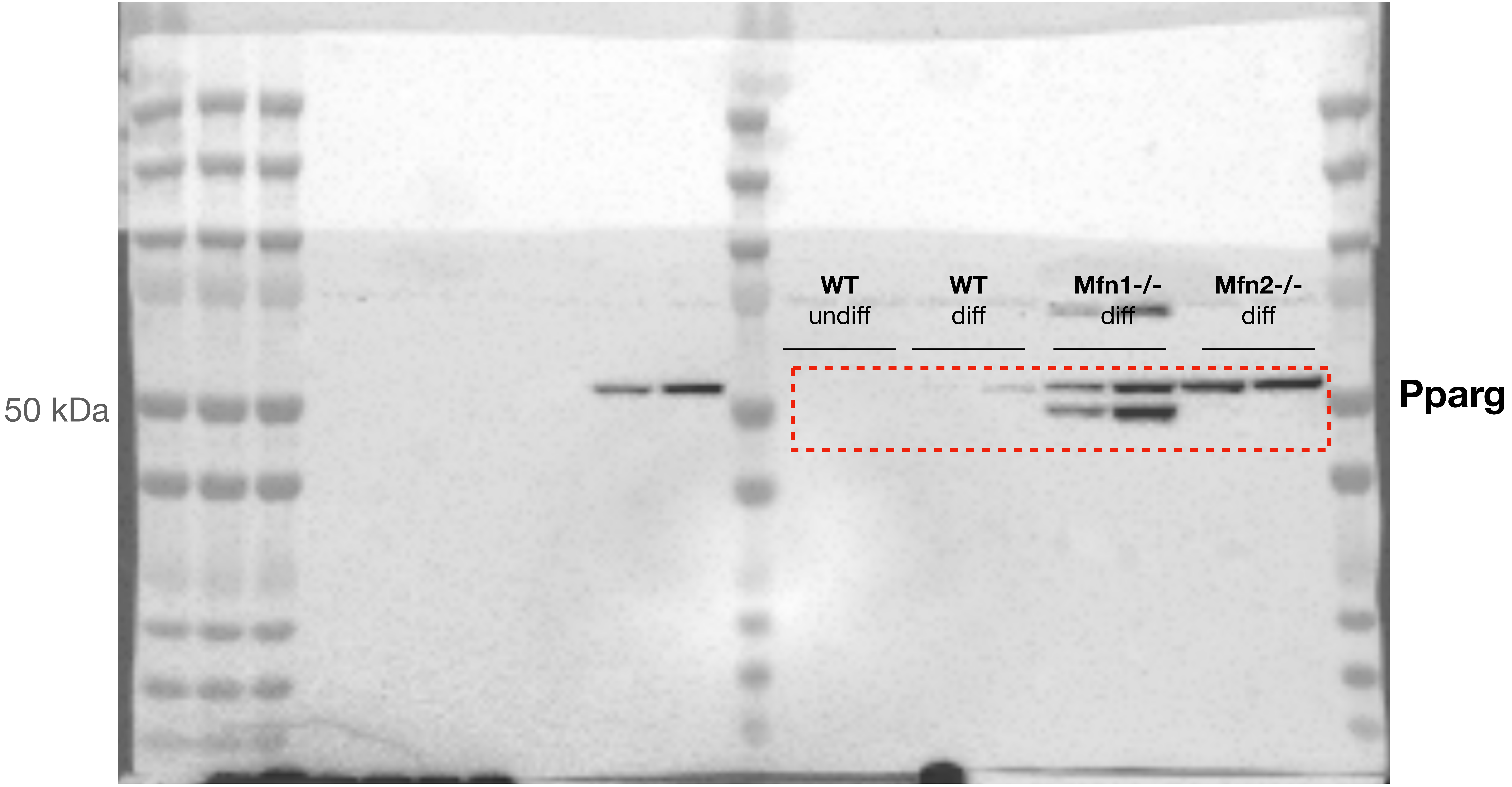

S3 Figure E

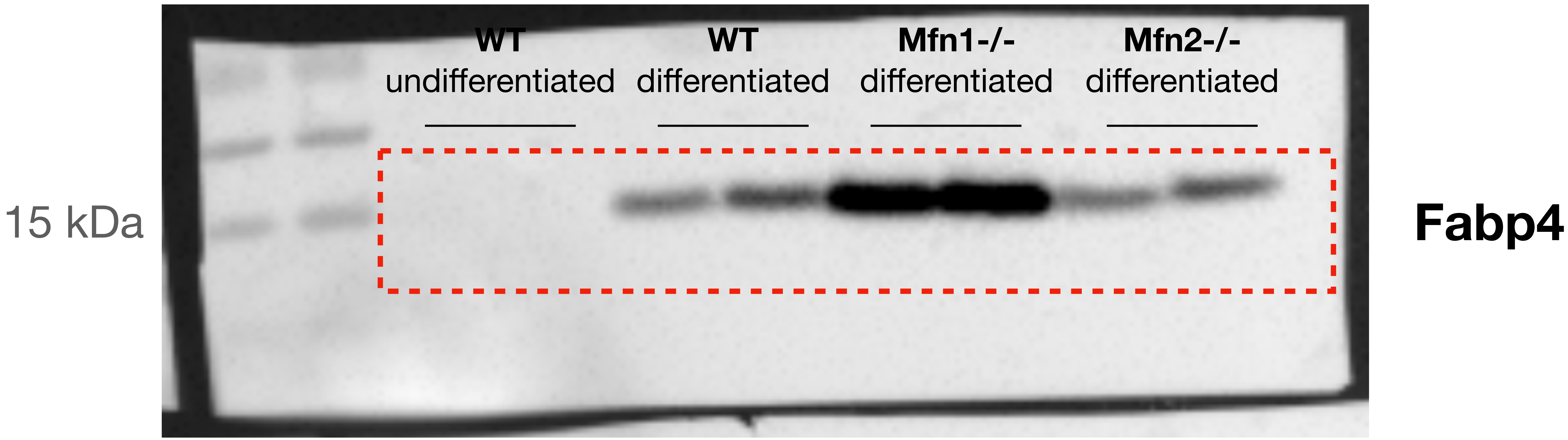

WT
undifferentiated
WT
differentiated
Mfn1-/-
differentiated
Mfn2-/-
differentiated
15 kDa
Fabp4

S3 Figure E

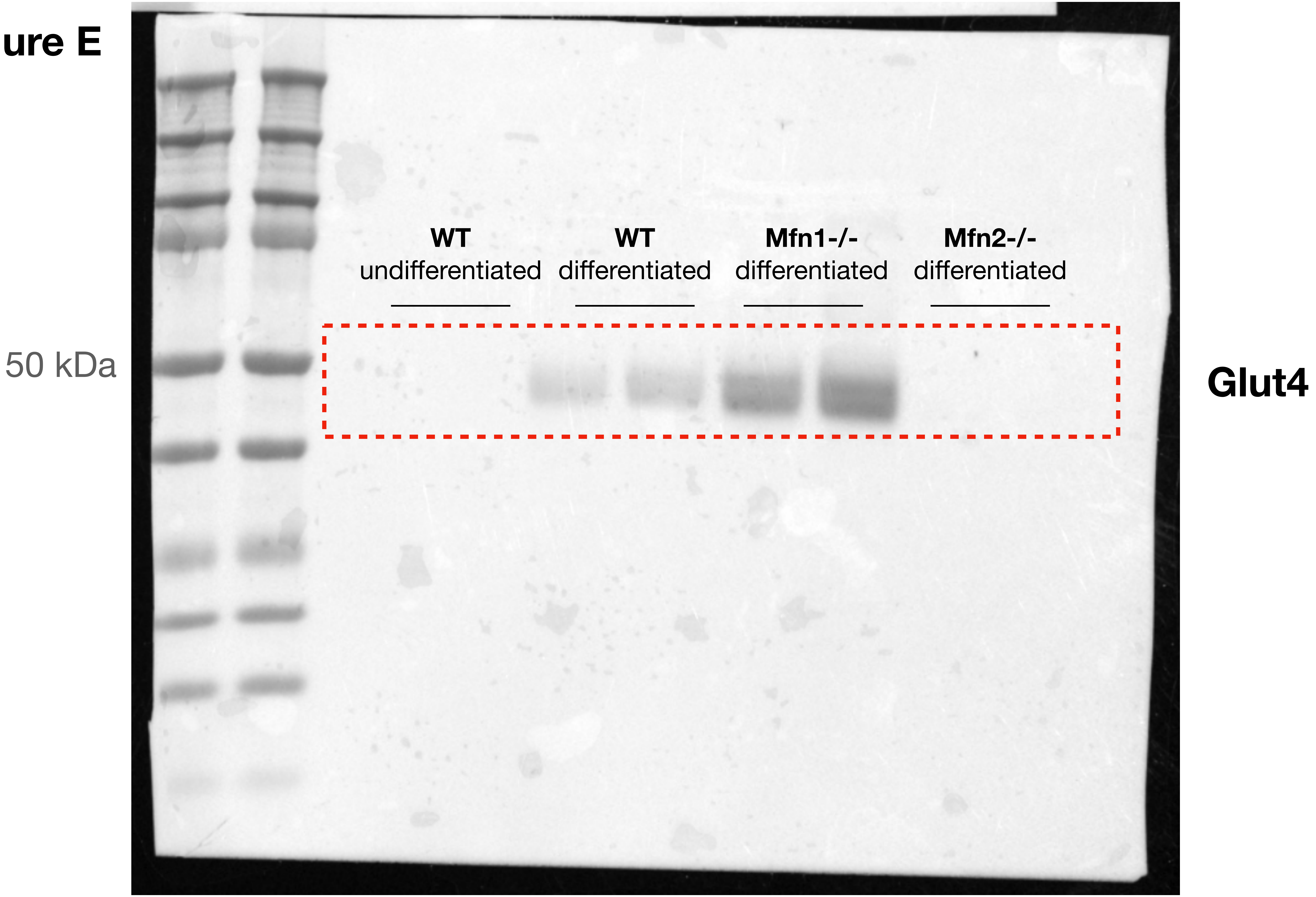
50 kDa
WT
undifferentiated
WT
differentiated
Mfn1-/-
differentiated
Mfn2-/-
differentiated
Glut4

S3 Figure E

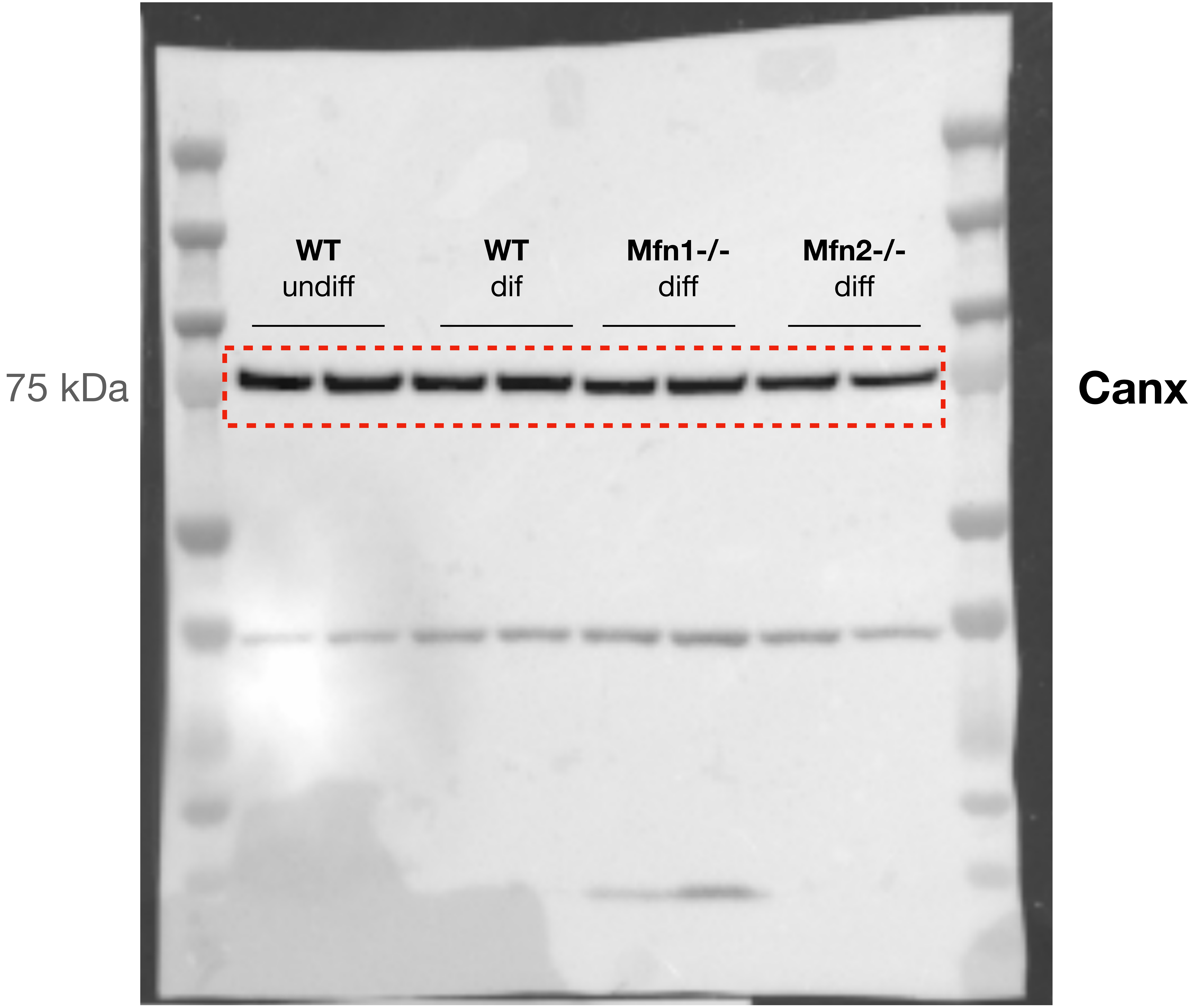

S6 Figure A

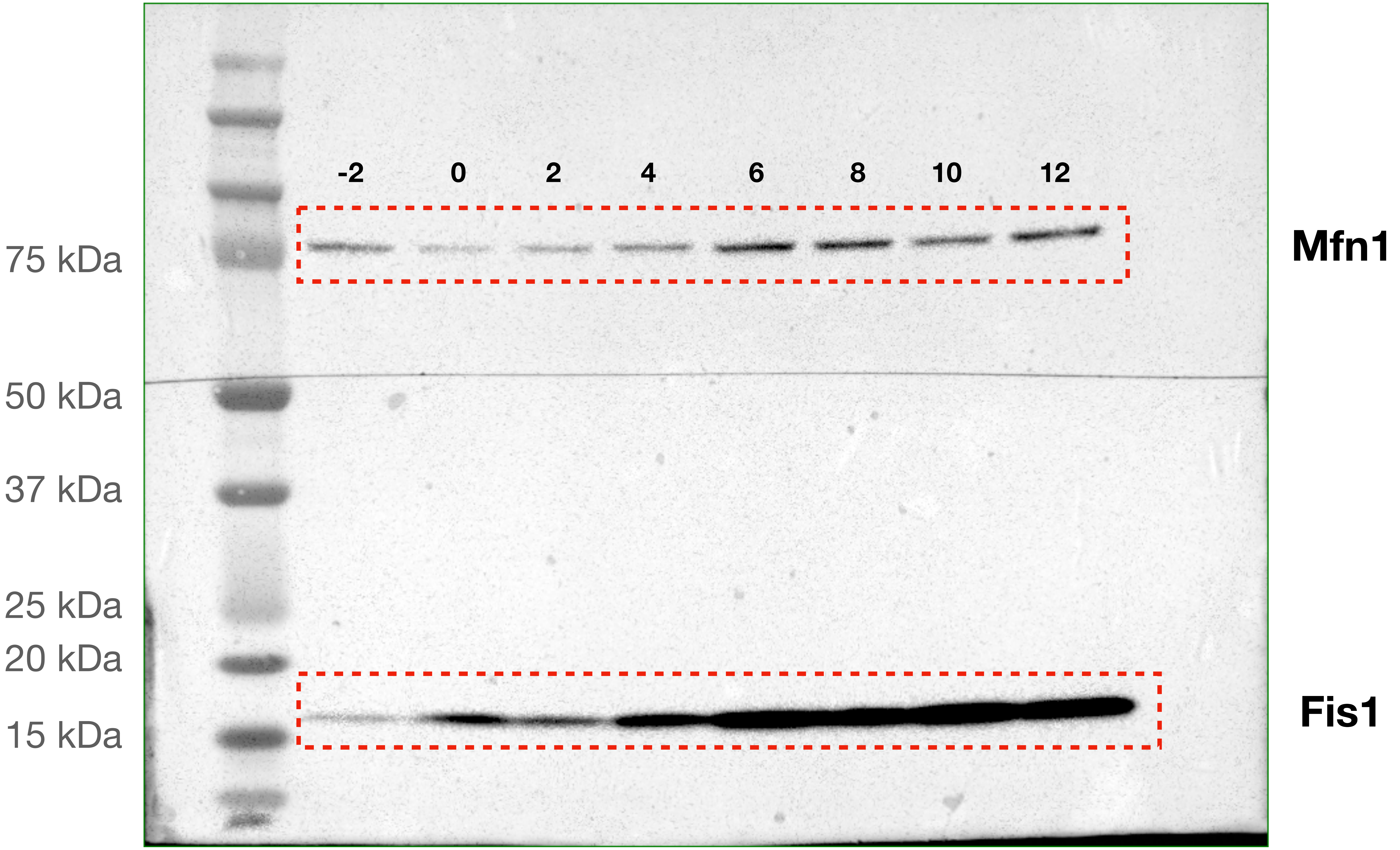

S6 Figure A

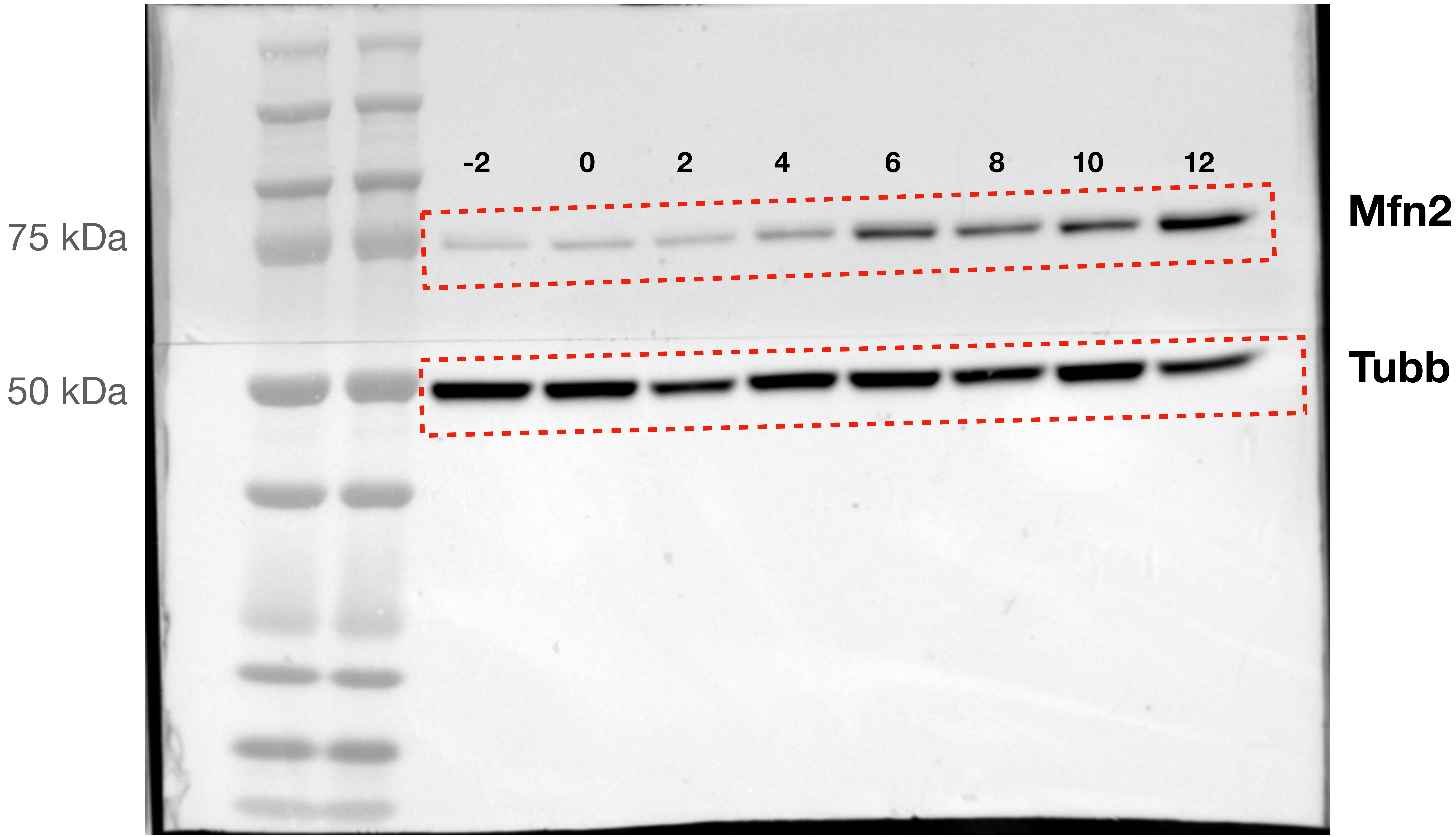

Figure 5A

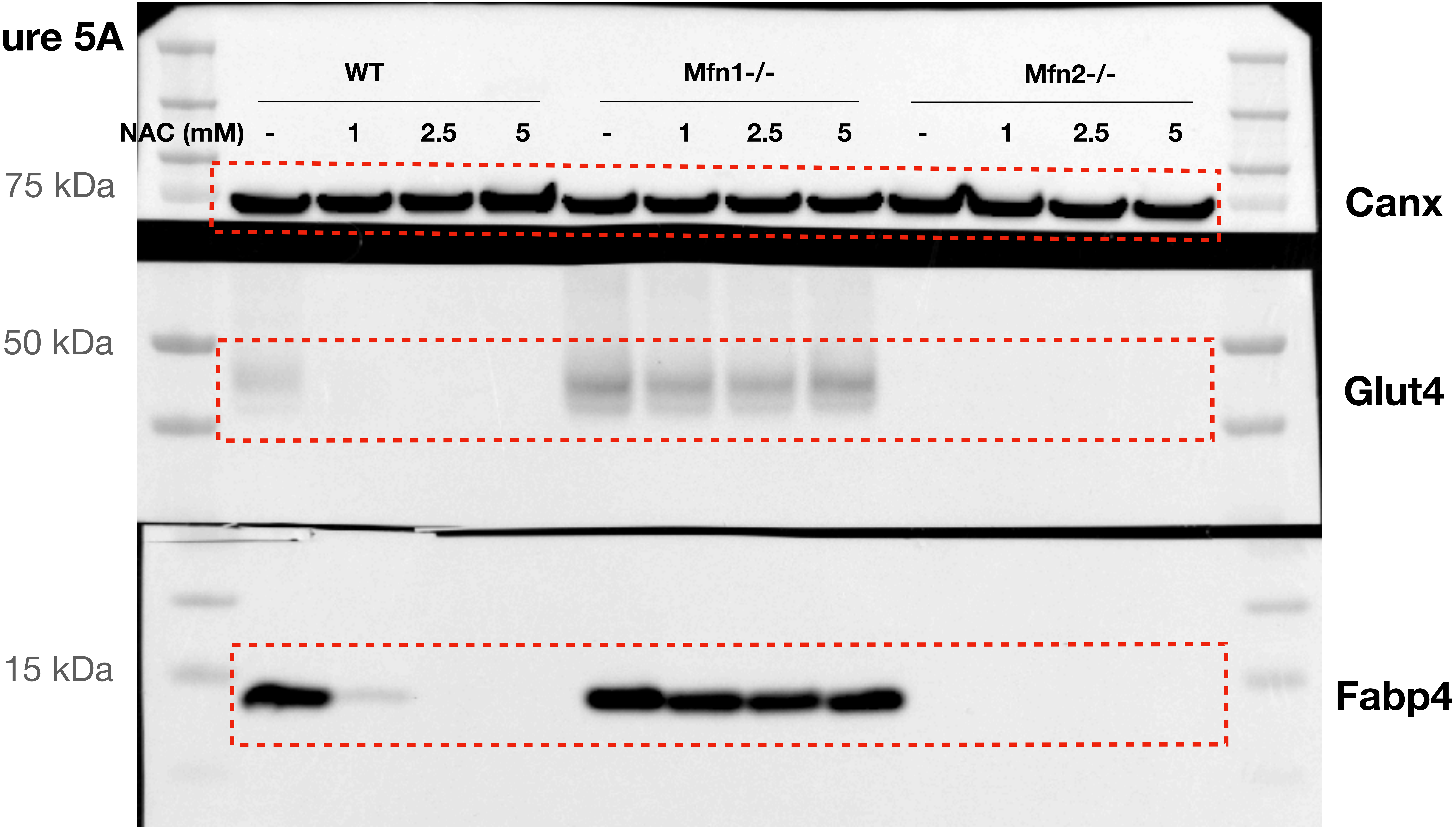
WT
Mfn1-/-
Mfn2-/-
NAC (mM)
- 1 2.5 5 - 1 2.5 5 - 1 2.5 5
75 kDa
50 kDa
15 kDa
Canx
Glut4
Fabp4

Figure 5A

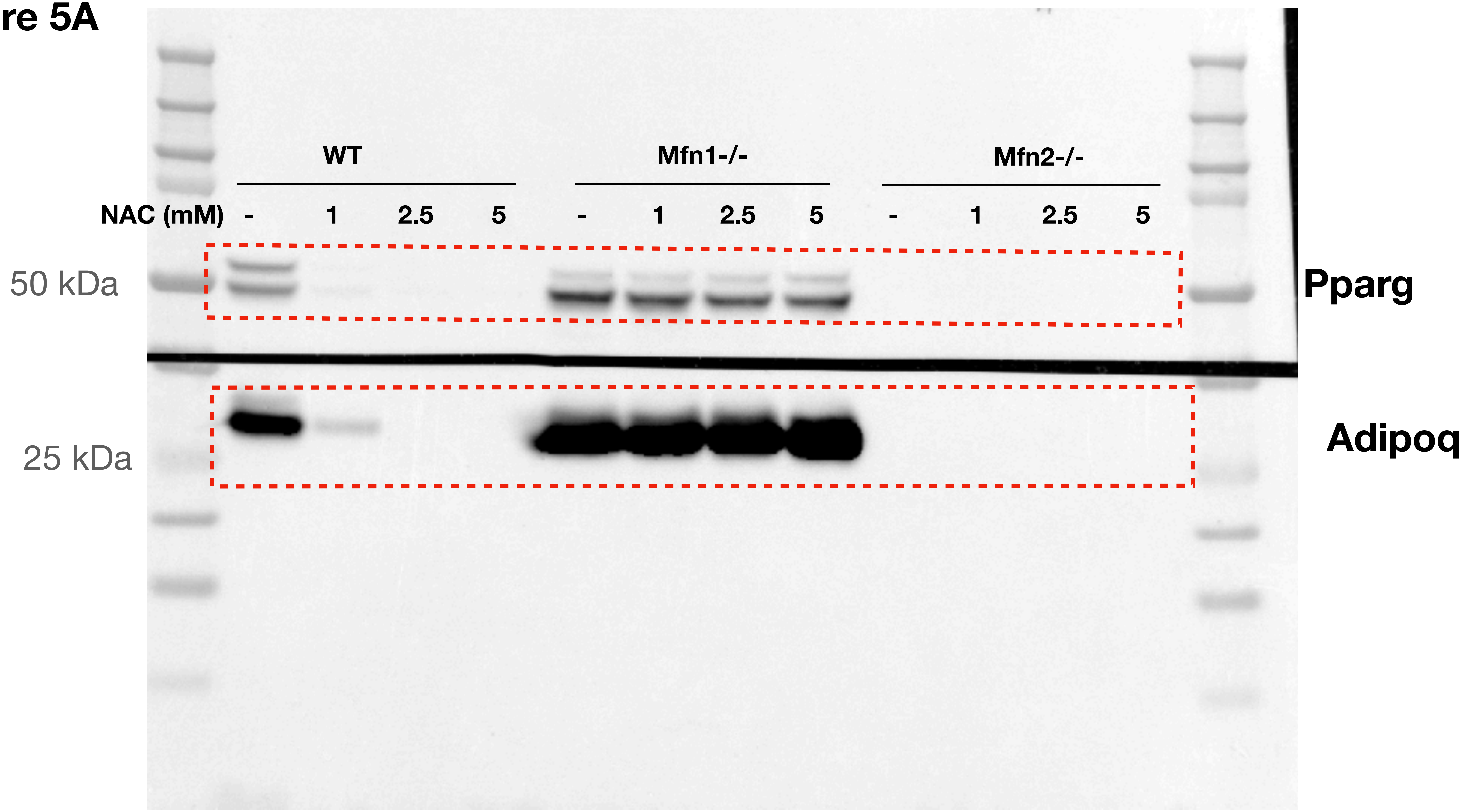

Figure 5A

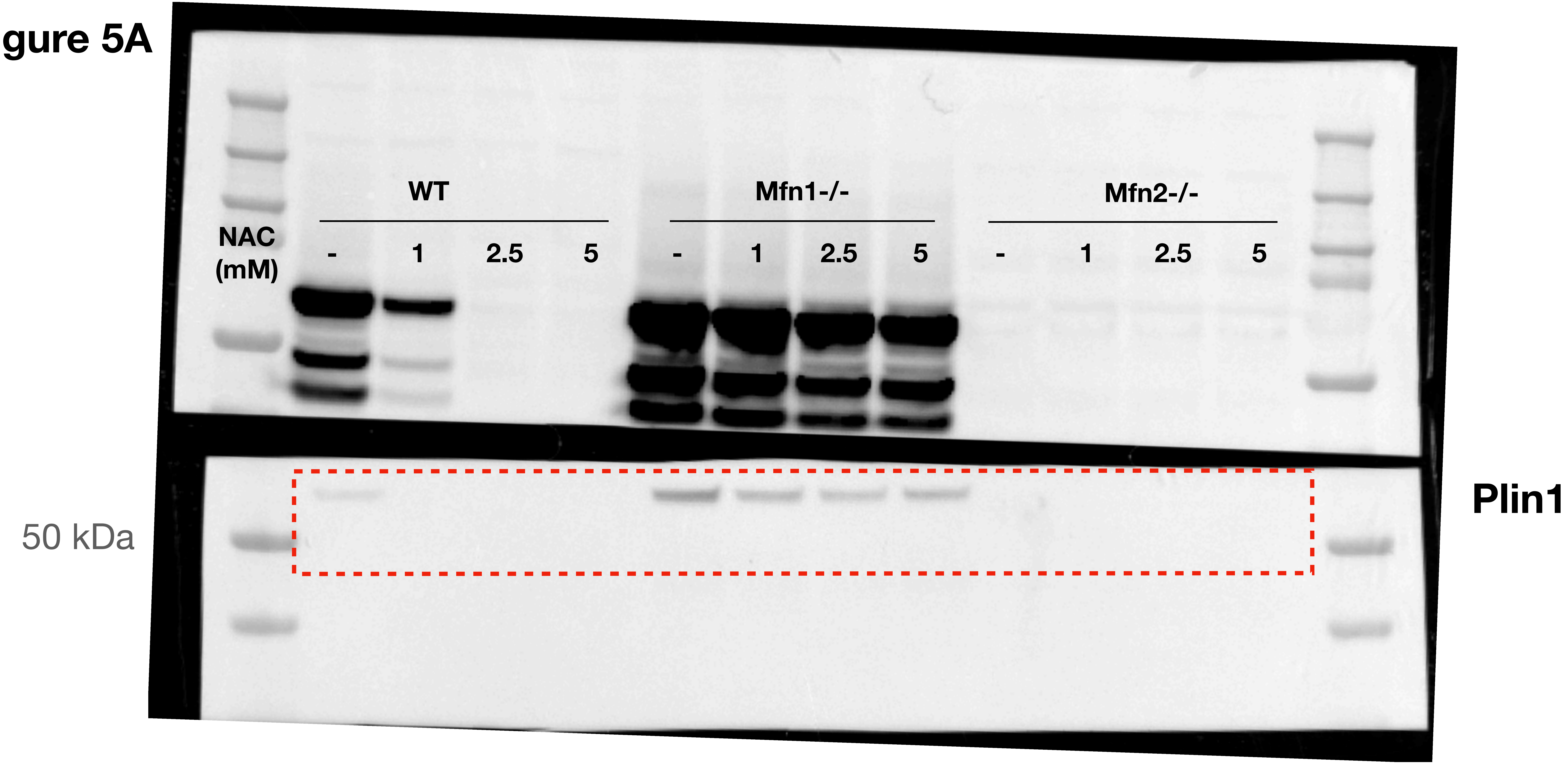

Supplement: S1 Raw images — (PDF) [file pone.0306243.s011.pdf]
