# Supplementary material for: Loss of Mfn1 but not Mfn2 enhances adipogenesis
Source: PLoS One. 2024 Dec 31;19(12):e0306243. doi: 10.1371/journal.pone.0306243 (PMC11687706; doi:10.1371/journal.pone.0306243)
Supplement: Attachment — Submitted filename: 131693_1_rebuttal_2682406_r6w62n.docx [file pone.0306243.s012.docx]

**Manuscript number:** RC-2022-01748

**Corresponding author(s):** David B. Savage

## 1. General Statements

Dear Dr. Monaco,

Thank-you for the opportunity to revise and resubmit this manuscript. We are also very grateful to the reviewers for their generally supportive comments and helpful suggestions.

Our responses to each of the points made are shown below. All authors have seen the revised manuscript.

We hope that the revised manuscript addresses your suggested revisions.

Yours sincerely

David

## 2. Point-by-point description of the revisions

Reviewer #1 (Evidence, reproducibility and clarity (Required)): 

Major points: 

1. Although the role of mitofusin on mitochondrial morphology has been established by others and comprehensively assessed in the present study, the authors should determine the functional outcome from the genetic manipulations on Mfn2 and Mfn1. As observed by increased glucose uptake, one could hypothesize an impairment in mitochondrial oxidative phosphorylation, leading the cells to rely uniquely or heavily on glycolysis as a fuel. Also, as mentioned by the authors in the discussion, ROS play a fundamental role in adipogenesis, and, therefore, mitochondrial ROS emission and/or cellular redox balance should also be assessed. I believe these two experiments will add insightful information to the current dataset.

**Thank you for these suggestions. Whilst we agree with the general premise of this point, unfortunately quantifying oxidative phosphorylation and ROS production with sufficient precision to detect relatively subtle changes remains very challenging. We have attempted these experiments but they require considerable optimisation (particularly using adipocytes). Preliminary studies done in MEFs (Cover letter figure 1) suggest that under some stimuli there may be higher ROS in Mfn1 and Mfn2 knock-out lines. However this preliminary data would require further optimisation and repetition in adipocytes, which is more challenging. For now, we have amended the Discussion to specify that these experiments are of particular interest.**

***Cover letter figure 1.*** *Levels of reactive oxygen species (ROS) in mouse embryonic fibroblasts measured by flow cytometry for fluorometric dyes CellROX (total cellular ROS), D2-HDCFA (total cellular ROS), and MitoSOX (mitochondrial ROS). Levels are expressed relative to wild-type. MEFs were treated with antimycin A (or media only) for 20minutes prior to incubation with the ROS dyes, then washed three times before assayed. AntA, Antimycin; CR, CellROX; M1, Mfn1-/- MEFs; M2, Mfn2-/- MEFs; MS, MitoSOX; WT, wild-type.*

2. The insulin effect on glucose uptake does not allow to conclude any impairment in insulin responsivity. The fold change of glucose uptake mediated by insulin was roughly 1.2 in undifferentiated adipocytes, 2.3 in differentiated WT, and 2.5 in Mfn1KO differentiated adipocytes. The absolute increase in glucose uptake could be a compensatory mechanism due to impairment in mitochondrial bioenergetics (see point #1), given that the cells can still respond to insulin. Measuring Akt phosphorylation levels following insulin treatment would help solve this issue.

**As requested, we have assessed the effect of insulin treatment on Ser 473 phosphorylation of *Akt2* (Pkb) in wild-type and knock-out MEFs differentiated into adipocytes (Fig 2D). Mfn1^-/-^ MEFs show an increase in Akt phosphorylation relative to the other cell lines. They also have higher expression of insulin receptor and Glut4, consistent with their degree of adipogenic differentiation.**

**We agree that impaired mitochondrial bioenergetics could account for the observations in perturbed glucose uptake in the knockout cell lines (especially Mfn2-null) and have therefore amended the text throughout to reflect this.**

3. Usually, working with clonal transgenic cells lines has the limitations that the cells might behave differently in terms of adipogenic potential over passages. A transient loss of function in the same cells would solve this concern. Also, introducing the patient mutations might be closer to the human situation than working with KO mouse fibroblasts.

**We agree with this potential concern, which is why we conducted knock-down studies in 3T3-L1 cells in addition to the work in knockout MEFs. These data were concordant with what we observed in the KO MEFs so we don’t think it is necessary to conduct repeat KD experiments in WT MEFs.**

**In our previous study we observed that human fibroblasts with biallelic MFN2-R707W mutations did not have any obvious phenotype (**[**https://elifesciences.org/articles/23813**](https://elifesciences.org/articles/23813)**). We have separate work studying these mutations *in vivo* where we provide further characterisation of murine adipocytes harbouring Mfn2-R707W; this work is now published here: https://elifesciences.org/articles/82283**

Minor points: 

4. Although the authors mention in the introduction that the differentiation of adipocytes is followed by an increase in mitochondrial mass, it would be interesting the determine the expression profile of mfn1 and mfn2 during the differentiation process.

**We have found that there is an increase in markers of mitochondrial fusion (Mfn1 & Mfn2) as well as fission (Fis1) throughout differentiation of 3T3-L1s. We have included this data in the manuscript (Supplementary Figure 6A**	**).**

5. The authors should discuss other models, even though pre-clinical, of mitochondrial dysfunction that results in lipodystrophy but with different metabolic outcomes. To cite a few but not only PMID: 29588285; PMID: 21368114; PMID: 31925461.

**Thank you for this suggestion. We have added a section on this in the introduction.**

6. It would be interesting to discuss the role of Mfn1/2 in the context of cold-induced adipogenesis, given the prominent role of mitochondrial dynamics, as mentioned by the authors in the reference list, on cold-induced adaptative thermogenesis (Mahdaviane et al. 2017; Boutant et al. 2017).

**Thank you for this suggestion. We have added a section on this in the introduction.**

Reviewer #2 (Evidence, reproducibility and clarity (Required)): 

- In Fig.2A, the authors report "increased lipid accumulation in Mfn1-/- MEFs, but not in Mfn2-/- MEFs". While the overall content might be similar, the pattern of lipid accumulation seems to be different. Indeed, differences in lipid droplet morphology have been observed in Mfn2 KO MEFs upon oleate treatment (McFie et al., 2016). The manuscript would benefit from having quantifications of lipid droplet size and number.

**Thank you for highlighting this. We have quantified lipid droplet size and, consistent with McFie et al have found increased size in Mfn2 knock-down. This data is now included in Supplementary Figure 6B.**

- Following the above point, McFie et al. also reported that Mfn1/Mfn2 double KO MEFs could differentiate into adipocytes. The authors should discuss these opposing observations. 
**The contrasting observation may be due to acquired clonal differences in MEF lines. We have attempted ‘double’ knock down (of both Mfn1 and Mfn2 concurrently) in 3T3-L1 cells however this was essentially lethal and also did not generate any cells capable of differentiation. We have added a section in the Discussion regarding this point.**

- In relation to the effects of Mitofusin deletions on glucose uptake, the authors mention that Mfn2 KO MEFs show impaired insulin stimulated glucose uptake. The interpretation of the result is not straight forward, as basal glucose uptake is highly increased in Mfn2 KO MEFs. Maybe there is simply a treshold for maximal glucose uptake capacity in MEF-derived adipocytes. In any of these cases, the authors might want to check GLUT1 levels, in line of their suggestion that the increased basal glucose uptake might be related to higher GLUT1. Alternatively, the authors might also want to check elements of the insulin signaling path, in case there are alterations that could explain the phenomenon.

**As mentioned above in response to reviewer 1, we have now performed immunoblots to quantify some components of the insulin signalling cascade (Fig 2D). We observed lower expression of both Glut1 and Glut4 in the Mfn2-/- cells. Mfn2-/- cells did demonstrate some Akt phosphorylation but considerably less than Mfn1-/- cells. These results are now included in the revised manuscript (Figure 2D).**

- In line with the above point, one would have wished that mitochondrial biology was better characterized in the different MEF models. While mitochondrial shape analyses are provided, some information on, at least, mitochondrial respiratory capacity, glucose oxidation and/or fatty acid oxidation rates, would be important. This would allow for a more solid discussion on why Mfn2 KO MEFs display such high basal glucose uptake rates.

**We have responded to a similar suggestion from Reviewer 1, above.**

- In relation to the experiments in MEFs, one should never forget that WT, Mfn1 and Mfn2 KO MEFs derive from different mice. Hence, the phenotypes could be related to trait variabilities in the origin mice themselves, and not just the gene deletion. To control for this aspect, the authors could simply re-introduced Mfn1 or Mfn2 in their respective MEFs and evaluate if their alterations are normalized.

**Yes one could try this but we have addressed this general concern by replicating the impact of Mfn1/2 KD in 3T3L1 cells so are not inclined to pursue this at this time.**

- Transcriptomic analyses reveals a decrease in adipogenic gene expression in Mfn2 KO MEFs. However, lipid accumulation is comparable to WT MEFs is normal. This could be due to defects in lipolytic capacity, leading to similar lipid accumulation despite lower adipogenic capacity. This could be tested by evaluating the adrenergic response of these cells (e.g.: glycerol release).

**Thank you for this suggestion. We have commented in the Discussion to explain that we have not fully characterised this mechanism.**

- The experiments in 3T3-L1 would also benefit from some gene expression analyses to evaluate if Mfn1 depletion leads to acceleration and/or magnification of the differentiation stages. In relation to this, 3T3-L1 cells could be used to monitor Mfn1 and Mfn2 through differentiation, which in itself would be valuable information.

**We have performed a protein-level time course for markers of mitochondrial fusion (Mfn1 & Mfn2) as well as fission (Fis1) throughout differentiation of 3T3-L1s. We have included this data in the manuscript (Supplementary Figure 6A). We think that changes in protein expression are more relevant than changes in mRNA so have not included gene expression changes at this time.**

CROSS-CONSULTATION COMMENTS 
The comments from the three independent reviewers are extremely well aligned and agree that improving the following aspects could largely benefit the manuscript: 

- A better metabolic characterisation of the models used 
- Provide measurements in relation to mitochondrial bioenergetics and ROS production **– we have attempted this but the data is not very clear in our view and warrants further optimisation which we are not inclined to pursue currently.**
- Explorations of insulin signaling  - **done thank-you.**
- Improve the validation and significance of the cellular models used, following the different suggestions from the three reviewers. Most notably, considering the introduction of human Mfn2 mutation forms **– we have published a separate manuscript on follow up work on the human MFN2 variant as mentioned above.**

A number of additional comments are raised, all of which are very reasonable and, in my opinion, should not be difficult to address. I think we can all agree that a mechanistic underpinning of the observations would give a larger degree of novelty to the work. Also, none of us would like the revision's quality to be constraint by a tight deadline. I would therefore be totally OK to extend the timeframe for the revision beyond the original 3 months proposed. 

Reviewer #2 (Significance (Required)): 

This is an interesting and well-crafted manuscript. Mice deficient for Mfn2 or Mfn1 have been reported by different laboratories, yet most of them fail to explore the effects on early adipogenesis. The study is limited to cultured cells, but this is well acknowledged by the authors Given the existence of human mutations in the mitofusin-2 gene that largely alter fat mass distribution, this work provides new clues on how these mutations might impact adipose tissue. 


Reviewer #3 (Evidence, reproducibility and clarity (Required)): 

Mann et al. The objective of this study is to determine the extent to which mitofusins (Mfn1 and Mfn2) have redundant functions and assess their contributions to adipocyte differentiation. While a point mutation in the Mfn2 gene has been associated with severe adipose tissue dysfunction and lipodystrophy, no disease phenotypes have been linked to mutations in Mfn1. To address these objectives, the authors sought to characterize how adipocyte differentiation and function is affected in Mfn1, Mfn2 or double knockout adipocytes in two distinct in vitro models. Their findings indicate divergent effects of Mfn1 and Mfn2 on adipocyte differentiation and function despite similar alterations to mitochondrial morphology. Loss of Mfn1 promotes adipogenesis while Mfn2 decreases it. The authors conclude that these findings are indicative of non-redundant functions in Mfn1 and Mfn2. 

Major comments: 
The observation that Mfn1 KO/KD leads to increased adipogenesis in vitro is somehow novel and, perhaps, surprising, as the author say. However, the molecular understanding underlying this phenotype remains unexplored. The analyses performed are mainly descriptive and don't dig deeper into the identification of the molecular mechanism. They do hypothesize that ROS production may be responsible for the observed effects, but that's how far they go. 

The authors do highlight the limitations of this work, but these limitations need careful consideration, for not addressing them seriously limits the novelty of this study, especially not testing these conditions in human cells. 
The current version of this work seems too preliminary to suggest useful experiments that could strengthen the study, since future analyses could take many different directions.

**Yes, we accept that the findings are rather preliminary but our initial efforts suggest that precisely elucidating the underlying mechanism/s is likely to be more difficult and complicated than alluded to by the reviewers. We would therefor prefer to share our initial observations so that others can also attempt to clarify the underlying mechanisms.**

A few unanswered questions that the authors might consider are: 
What is the difference between the Arg707Trp mutation and the KO/KD? 
Mfn1 and 2 deletions lead to fragmented mitochondria, but opposite adipogenic potentials. What other mitochondrial defects can explain it? Are organelle contact site disrupted only with Mfn2? How does Mfn1 and 2 KO/KD affect mitochondrial proteome? 
What does mitochondrial bioenergetics look like? How is ROS production affected? 
Is the increased glucose uptake (basal) a compensatory mechanism for mitochondrial dysfunction? 
**Thank you for these suggestions. We acknowledge that this work is largely descriptive in nature. These are all questions that should be addressed to improve mechanistic understanding of our observations.**

**The difference between p.Arg707Trp and KO/KD is challenging to address because in the non-adipose cell lines studied so far (human and mouse fibroblasts) there has been no evidence of perturbation of the mitochondrial network.**

**As discussed above, we have done preliminary studies into ROS production but are unable to provide a complete characterisation at this time. Similarly, we have not been able to perform bioenergetic studies (e.g. Seahorse, Oxyboros) that would provide more insight into differences between Mfn1 and Mfn2 KO cell lines.**

CROSS-CONSULTATION COMMENTS 
I agree the work is interesting, but is too preliminary and merely descriptive. the experiments suggested will significantly improve the manuscript. However, I don't think they will take only three months to be completed. This work needs a significant amount of work including the study of the mechanism, at least an idea of what the mechanism could be, to be considered novel.

**We accept this limitation and have responded to this general point above.**

Reviewer #3 (Significance (Required)): 

Understanding how mitochondrial dynamics affect adipogenic differentiation is critical to better understand how metabolism impact cell signaling, cell fate and function. 

Strengths: this work reveals an interesting phenotype for Mfn1 and Mfn2 mutant preadipocytes. 
Weaknesses: this work is merely descriptive and preliminary to provide a clear understanding of the observed phenotypes 

Advance: Although the performed experiments are accurate, well designed, and well controlled, the fact that Mfn1 and 2 have distinct functions and cannot compensate for one another was already clear based on the embryonic lethality of either Mfn1 and Mfn2 KO mice as well as the Mfn2 mutation in humans that leads to a pathological condition.In the current verison, this work minimally contributes to advancing the field. 

Audience: an extensively revised version of this work including deeper phenotyping of thier models and human cell work would be of interest for sceintists studying mitchondrial biology, adipose tissue, metabolic diseases, and human genetic diseases.
