# Supplementary material for: Loss of Mfn1 but not Mfn2 enhances adipogenesis
Source: PLoS One. 2024 Dec 31;19(12):e0306243. doi: 10.1371/journal.pone.0306243 (PMC11687706; doi:10.1371/journal.pone.0306243)
Supplement: Attachment — Submitted filename: MFNAdipogen_Rev3_letter_May24.docx [file pone.0306243.s014.docx]

Dear Dr. Monaco,

Thank-you for the opportunity to revise and resubmit this manuscript. We are also very grateful to the reviewers for their helpful suggestions.

Our responses to each of the points made are shown below. All authors have seen the revised manuscript.

All original, uncropped blots are available in Supporting Information.

We hope that the revised manuscript addresses your suggested revisions.

Yours sincerely

David

-----------

Reviewer #1: The reviewer's comments have been appropriately addressed in general. However, the effects of the Mfn1 siRNA (01) and (02) are different without explanation (Rebuttal letter Figure 1). siRNA02 does not affect the expression of Plin1 and Adipoq and has minimal effect on Glut4 as compared to siRNA1. Is it due to different knockdown efficiency? The knock efficiency of the 2 siRNAs needs to be included and the rebuttal letter Figure 1 to be presented in the supplementary data.

Thank you for your suggestions. Yes, the differences observed are likely related to the relative knock-down efficacy. In optimisation, we tested three separate siRNAs, one showed very little efficacy and was not taken forwards. The other two (siRNA01 and siRNA02) demonstrated enhanced adipogenesis correlating with their relative ability to reduce expression of Mfn1.

These data have been included as Supplementary Figure 7, including a western blot showing the knock-down efficacy.
